# Intricacies of frustrated magnetism in the Kondo metal YbAgGe

**DOI:** 10.1038/s43246-026-01230-w

**Published:** 2026-06-27

**Authors:** D. G. Mazzone, C. B. Larsen, B. Ueland, X. Boraley, D. M. Pajerowski, Y. Skourski, J. Taylor, B. Fåk, S. L. Bud’ko, R. McQueeney, P. C. Canfield, O. Zaharko

**Affiliations:** 1 PSI Center for Neutron and Muon Sciences, 5232 Villigen PSI, Switzerland; 2 Department of Physics and Astronomy, Iowa State University, Ames, Iowa 50011 USA; 3 Ames National Laboratory, Iowa State University, Ames, Iowa 50011 USA; 4 Neutron Scattering Division, Oak Ridge National Laboratory, Oak Ridge, Tennessee 37831 USA; 5 Dresden High Magnetic Field Laboratory (HLD-EMFL), Helmholtz-Zentrum Dresden-Rossendorf, 01328 Dresden, Germany; 6 ISIS Facility, STFC Rutherford Appleton Laboratory, Harwell Science and Innovation Campus, Oxfordshire OX11 0QX, UK; 7 Institut Laue-Langevin, CS 20156, 38042 Grenoble Cédex 9 France

**Keywords:** Magnetic properties and materials, Electronic properties and materials, Quantum physics

## Abstract

The combination of localized magnetic moments, their frustration and interaction with itinerant electrons is a key challenge of condensed matter physics. Frustrated magnetic interactions promote degenerate ground states with enhanced fluctuations, a topic that is predominantly studied in magnetic insulators. The coupling between itinerant and localized electrons in metals add complexity to the problem, and is presently formulated only for extreme cases in which the itinerant electrons mediate exchange between localized spins (RKKY interaction) or suppress the formation of magnetic moments (Kondo screening). Here, we report an in-depth experimental study of the distorted Kagome metal YbAgGe, unravelling the open questions of how frustration, localized magnetism and itinerant electrons are intertwined in frustrated Kondo metals. We find that coupled itinerant and localized electrons give rise to dynamic magnetic correlations below T^*^ ≈ 20 K. At lower temperature, frustrated magnetic interactions establish anisotropic magnetic short-range correlations that culminate into antiferromagnetic long-range order below *T*_*N*_ = 0.68 K with a significantly reduced modulated magnetic moment. We show that local moment Hamiltonians can yield limited understanding of the microscopic behaviour in frustrated metals, and prompt the extension of more sophisticated model Hamiltonians incorporating itinerant effects.

## Abstract (web summary)

The interplay of localized magnetic moments, frustration, and itinerant electrons in metals remains a complex challenge in condensed matter physics. Here, the authors investigate the distorted Kagome metal YbAgGe, revealing dynamic magnetic correlations and anisotropic short-range order, prompting advanced models to better understand frustrated Kondo metals.

## Introduction

Magnetic frustration in materials arises from constraints of the underlying lattice geometry or conflicting exchange interactions, which can yield degenerate magnetic configurations with unusual ground states such as skyrmion lattices^1^, spin glasses^2^, spin liquids^3^, and other phases that may be attractive for future technological applications. A microscopic understanding of these frustrated magnetic states is often achieved through local-moment magnetic Hamiltonians with nearest-neighbour spin interactions^4,5^. These microscopic models have proven fruitful for insulating materials in which itinerant electrons can be neglected, further neighbour interactions decay promptly with distance, and higher-order spin interactions are strongly suppressed^6^. The approach is less effective for magnetically frustrated metals. For this material class, it is often necessary to account for long-range Fermi surface effects, in which conduction electrons interact with the local moments of the magnetic ions.

Microscopic Hamiltonians appropriate for magnetic metals with embedded localized moments are derived from the Kondo lattice Hamiltonian (KLH) *H*_*KLH*_ = *H*_*kin*_ + *H*_ex_^6,7^. Here, *H*_*kin*_ describes the kinetic motion of the itinerant electrons, and *H*_*ex*_ represents the coupling of the localized spins with the conduction electrons. The KLH is particularly important for rare-earth compounds^8^, for which the relevant RKKY interaction^9–11^ can be derived as lowest-order contribution^6^. In this model, the conduction electrons serve as exchange pathways between local magnetic moments, directly encoding the Fermi surface into local exchange parameters *J* of effective Heisenberg-like Hamiltonians. Within this approach, it is possible to treat magnetism in metals similarly to the insulating case, but with anisotropic exchange interactions. This allows for the exploration of novel frustrated quantum phases in metals^6,12–15^, though their interpretation often remains challenging.

Metals containing rare-earth ions such as Ce, Eu, Sm, and Yb experience an additional interaction. The conduction electrons can (partially) screen localized moments, engaging them into collective singlet states^16^. This Kondo screening process can be understood through an extension of *H*_*ex*_, encrypting an antiferromagnetic coupling between the localized *f*-electrons and the conduction bands^17^. The admixture between these electrons can become coherent at low temperature, leading to hybridized electron bands with heavy effective masses close to the Fermi surface. A microscopic band description of these heavy-fermion systems has proven to be notoriously challenging, as most ab-initio calculations have difficulties to distinguish the small energy differences of the various emergent states. Magnetic frustration complicates the matter, because theoretical predictions need to ascertain whether various spin configurations are degenerate or separated in energy. To this end, we address the question of how the three ingredients—RKKY interaction, Kondo screening, and frustration—intertwine to establish the physics in YbAgGe.

## Results

### Local magnetism and frustration

**Fig. 1:**
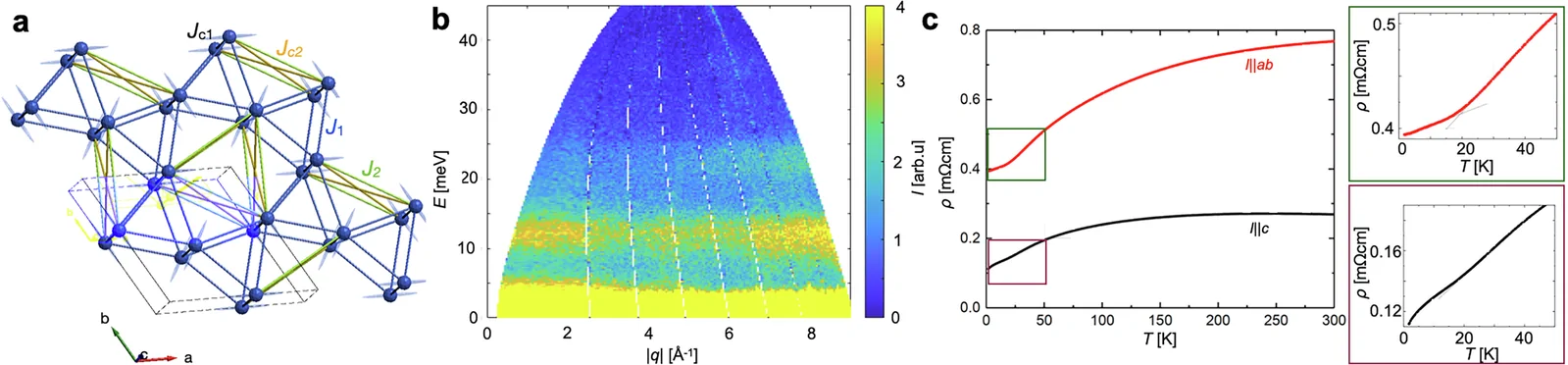
The three main ingredients in frustrated Kondo metals. **a** *Frustration:* the Yb^3+^ ions in YbAgGe generate a distorted Kagome structure with nearest and next-nearest neighbour interactions *J*_1_ (blue) and *J*_2_ (green) in the hexagonal plane and nearest neighbour *J*_*c*1_ (black) and *J*_*c*2_ (gold) interplane interactions. Single ion anisotropy ellipsoids are shown in blue. **b** *Local magnetism:* inelastic neutron scattering data for energy transfers *E* < 45 meV and wavector transfers ∣*q*∣ < 9 Å^−1^ measured at *T* = 3 K on the MARI spectrometer. The transition at *E* = 12 meV originates from a CEF excitation mixed with a phonon, the transition at *E* = 23 meV is attributed to phononic contributions. **c** *Itinerant electrons:* electrical resistivity measurements in which the electric current *I* runs parallel or perpendicular to the crystallographic *c*-axis. Below T^*^ = 20 K additional scattering processes yield deviations from the temperature dependence at higher temperature. Note that the material is known to possess a high residual resistivity value that does not arise from crystalline disorder, but stems from scattering processes which can be suppressed efficiently with magnetic field^24^.

YbAgGe is a member of the *R*AgGe family crystallizing in the hexagonal ZrNiAl-type crystal structure (space group $P\bar{6}2m$)^18^, in which Yb^3+^ ions form distorted Kagome layers with periodicity *a* = *b* = 7.05 Å perpendicular to *c* = 4.14 Å (see Fig. 1a and Supplementary Information (SI) Note 1 for details). Within the localized model the overarching magnetic energy scale is determined by the localized Yb^3+^ Kramers ions, with total angular momentum *J* = 7/2. The 2*J* + 1 = 8 degenerate eigenstates of the Yb^3+^ ion are split by the non-spherical crystal-electric field (CEF). The lowest CEF transition has at an energy transfer of *E* = 12.0(4) meV, manifesting as a non-dispersing excitation in our polycrystalline inelastic neutron scattering (INS) experiment on the MARI spectrometer at ISIS (Fig. 1b). The transition is in agreement with earlier reports^19,20^. Using PyCrystalField^21^ our calculation establishes that the ground state retains the axial anisotropy, aligned with 2-fold axes passing through the Yb^3+^ ions (see Fig. 1a) and predicts a magnetic moment of *μ*_*CEF*_ = 3.98*μ*_*B*_ (see Supplementary Information Note 2 for details).

Within the localized picture the Heisenberg exchange Hamiltonian approximation enables to predict effective magnetic models for the *R*AgGe crystal geometry. They range from Kagome spin layer models, when the frustration between intraplane interactions *J*_*i*_ prevails, to spin chain models, when the frustration is dominant within the interplane interactions *J*_*ci*_. For instance, in the *R* = Tm member the energy hierarchy can be resolved as follows^22^: (i) the CEF-anisotropy dominates the magnetic exchange interactions; (ii) the strongest magnetic exchange coupling *J*_*c*_ along the *c*-axis is ferromagnetic (FM) and not frustrated; (iii) the interplay between weaker, intraplane *J*_1_, *J*_2_ couplings manifests ice rules for the *J*_2_ triangles, but the spin-ice frustration is released by the significant equidistant but distinct *J*_3*a*_ and *J*_3*b*_ terms (see alsoSupplementary Information Note 3). In YbAgGe the CEF anisotropy is the same as in TmAgGe, but its contribution is less strong. This allows for interplane interactions *J*_*ci*_ to gain in importance, yielding frustration effects alongside the interplane couplings.

Macroscopic studies and earlier neutron diffraction results^23^ have reported that antiferromagnetic (AFM) long-range order (LRO) in YbAgGe is established below *T*_*N*_ = 0.68 K with a propagation vector of *q* = ($\frac{1}{3}$, 0, $\frac{1}{3}$). The order is unconventional, featuring varying moment sizes on different atoms with an average amplitude of 1.62(3)*μ*_*B*_/Yb. A magnetic field applied in the Kagome plane triggers a series of metamagnetic transitions to other LRO states with different periodicity in the Kagome plane, but with persistent ${q}_{z}\approx \frac{1}{3}$ component. Above *μ*_0_*H* ≈ 7 T (*H*∥ [1, −1, 0]), these modulations vanish, so that eventually only an induced FM component remains (see Supplementary Information Note 1 for details). We attempted to conceive the dominant exchange interactions in YbAgGe by minimizing the Heisenberg-type Hamiltonian and analyzing the solutions with the propagation vectors determined in earlier experiments^23^ (see Supplementary Information Note 3). We find that the *q* = ($\frac{1}{3}$, 0, $\frac{1}{3}$) propagation vector requires AFM nearest-neighbour interplane interactions *J*_*c*1_ and *J*_*c*2_, and finite further neighbour intraplane terms *J*_3_.

**Fig. 2:**
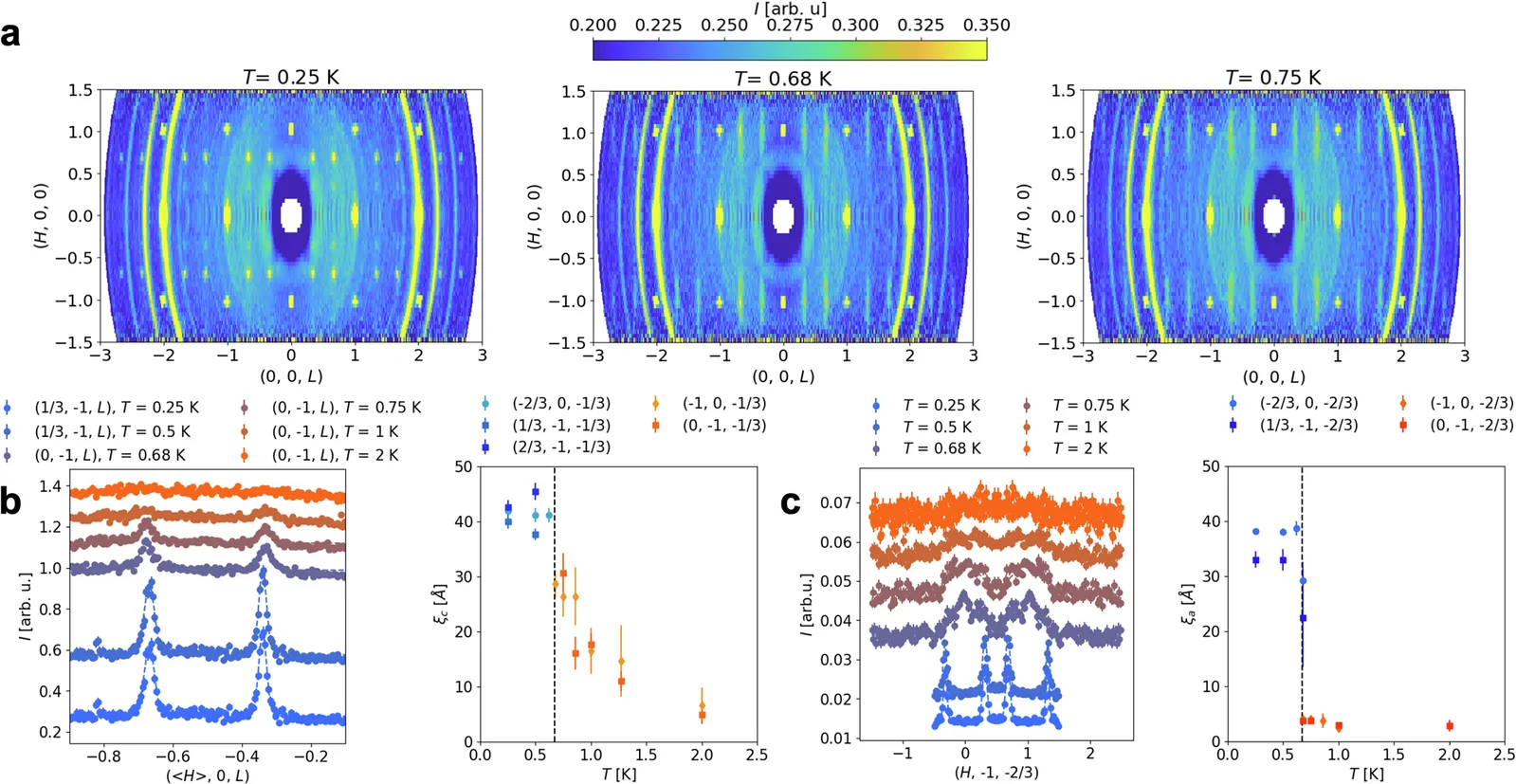
Static magnetism in YbAgGe. **a** Two dimensional reciprocal space map in the (*H*, 0, *L*) plane measured at *T* = 0.25, 0.68, and 0.75 K. Below *T* ≈ 2 K antiferromagnetic short-range correlations emerge at a wavevector *q* = (0, 0, $\frac{1}{3}$) with a correlation length along the *c*-axis which exceeds the in-plane correlation length. Below a first-order transition at *T*_*N*_ = 0.68 K long-range order is developed at *q* = ($\frac{1}{3}$, 0, $\frac{1}{3}$). The data were measured at the Cold Neutron Chopper Spectrometer (CNCS) using an incident energy of *E*_*i*_ = 12 meV. The figures were generated using an energy integration Δ*E* = [−1/2, 1/2] meV and a reciprocal space integration Δ*Q* = [−0.1, 0.1] rlu perpendicular to the reciprocal plane. **b** One-dimensional line-cuts and quantitative analysis of the short-range correlations in YbAgGe along the crystallographic *c*- and **c** *a*-direction. The four dimensional data was integrated over Δ*E* = ±0.25 meV around the elastic line and Δ*Q* = ± 0.05 rlu perpendicular to the shown cutting direction. The cuts are offset for clarity. The resulting one-dimensional line-cuts were fitted by Gaussian line shapes, from which the correlation lengths *ξ*_*c*_ = *c*/*π*FWHM and *ξ*_*a*_ = *a*/*π*FWHM were determined as function of temperature. FWHM denotes the full-width at half-maximum of the Gaussian peaks.

**Fig. 3:**
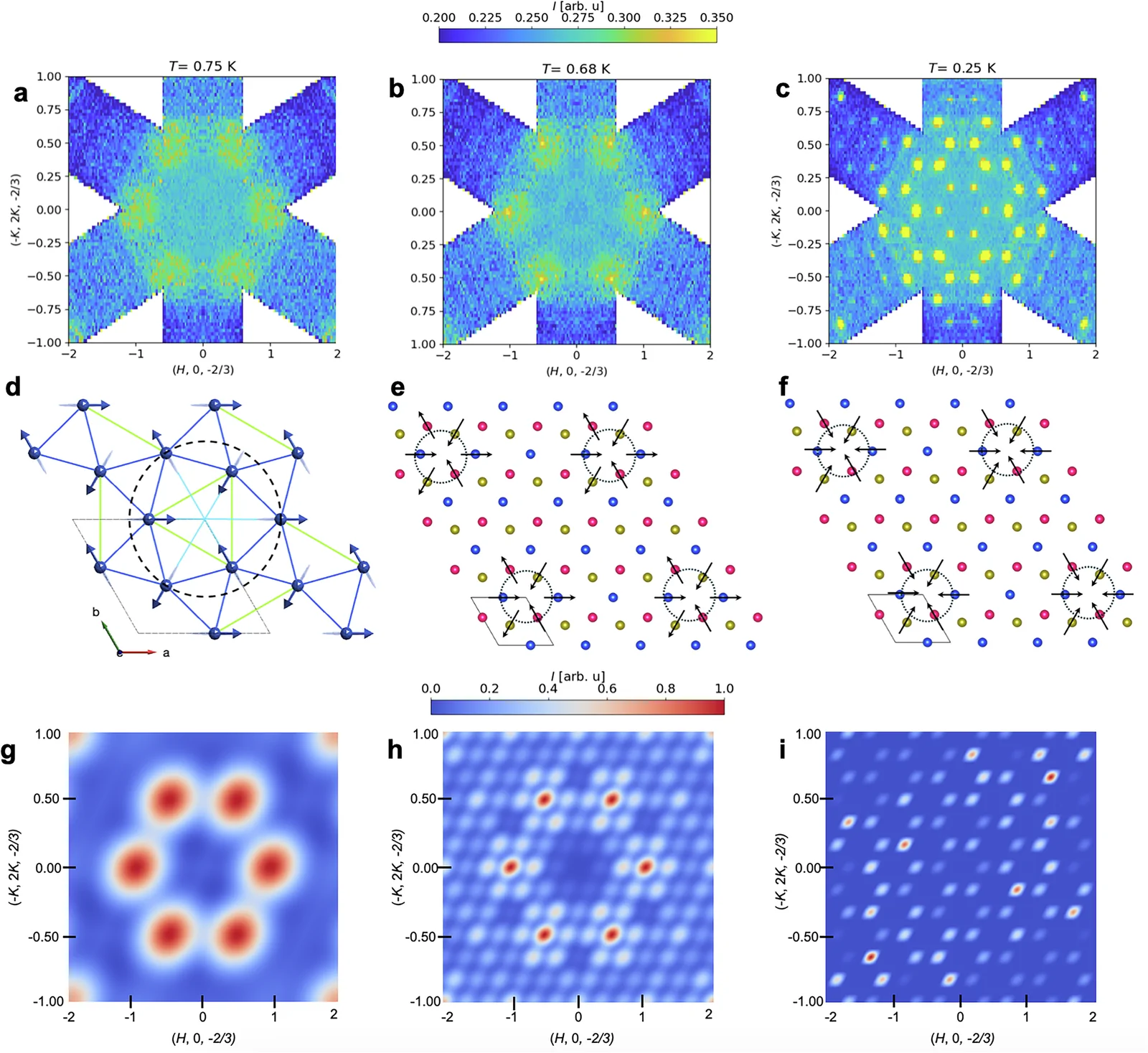
Static magnetism in YbAgGe. Two dimensional reciprocal space maps perpendicular to (0, 0, −2/3) measured at *T* = 0.75 **a**, 0.68 **b** and 0.25 K **c**. The data were measured at CNCS using an incident energy of *E*_*i*_ = 12 meV. The figures were generated using an energy integration *ΔE* = [−1/2, 1/2] meV and a reciprocal space integration *ΔQ* = [−0.69, −0.63] rlu perpendicular to the reciprocal plane. **d** The magnetic moment arrangement within the hypothetical *q* = (0, 0) structure, a dashed black circle spans over a Yb-hexagon. **e** The 3a^*^ - 3b^*^ structure with the parallel moment alignment connected by *J*_3*a*_ (labeled in Supplementary Fig. S3b). **f** The 3a^*^ - 3b^*^ structure with the antiparallel moment alignments. Calculated (*H*, *K*) cuts simulated via Fourier transform of **g** a 3 × 3 × *N* box containing the hypothetic *q* = (0, 0) structure, **h** a 10 × 10 × *N* box with the parallel moment alignment connected by *J*_3*a*_, **i** a 24 × 24 × *N* box containing three 120 deg rotated domains of the zero-field *q*_1_= ($\frac{1}{3}$, 0, $\frac{1}{3}$) amplitude modulated structure^23^ with parallel and antiparallel moment alignments connected by *J*_3*a*_.

Previous thermodynamic measurements have shown that YbAgGe exhibits increased magnetic correlations already above *T*_*N*_, for *T* < *T*_*SR*_ ≈ 2 K^24^. Here we reveal their microscopic origin via single crystal elastic scattering maps, which we measured on the Cold Neutron Chopper Spectrometer (CNCS) at ORNL. Figures 2a and 3a show elastic scattering maps in the (*H*, 0, *L*) and (*H*, *K*, −2/3) planes for *T* = 0.25, 0.68, and 0.75 K. Upon cooling below *T* ≲ 2 K diffuse scattering develops around the *q* = (0, 0, $\frac{1}{3}$) positions (see Fig. 2). The correlation lengths *ξ*_*c*_ and *ξ*_*a*_ show that at *T* = 2 K, the material is correlated over ~5 Å along the *c*-direction, and ~4 Å along the *a* direction. Upon cooling *ξ*_*c*_(*T*) is continuously increasing and reaches the resolution limited correlation of structural Bragg peaks (~10 unit cells) below *T* = 0.68 K, where magnetic long-range order with *q* = ($\frac{1}{3}$, 0, $\frac{1}{3}$) is established. This behavior is different for *ξ*_*a*_(*T*), where the correlation length remains unchanged above *T* > 0.68 K. A discontinuous jump of *ξ*_*a*_(*T*) is observed at *T* = 0.68 K below which the correlation length reaches 5 unit cells, matching the correlation length of structural Bragg peaks.

The diffuse scattering patterns correspond to spin-spin correlations that were modeled using fast Fourier transform and sampling theory to reduce the high-frequency noise in the calculations as implemented by the Scatty program^25^. Above *T*_*N*_ = 0.68 K YbAgGe is short-range ordered within the hexagonal plane but reveals extended correlations along the *c*-axis. Thus, we neglected the *J*_*cij*_ terms (see Fig. 1a) because the *q*_*z*_= $\frac{1}{3}$ correlations are already established at these temperatures. We first modeled the diffuse scattering at *T* = 0.75 K, for which we used a hypothetical two-dimensional long-range *q* = (0, 0) structure with 120 deg canted magnetic moments such as presented in (Fig. 3d). The order represents a compromise between the local CEF anisotropy and AFM *J*_1_ and *J*_2_ couplings; the moments connected by the *J*_3*a*_- and *J*_3*b*_- bonds are parallel within such an arrangement. The corresponding reciprocal *hk*-plane consists of sharp magnetic Bragg peaks at integer positions with *h* + *k* = 2*n* + 1. Magnetic correlations confined to a few unit cells result in diffuse scattering around these *hk* -positions, which is reproduced by a small number of unit cells along the *a*- and *b*-axes. Notably, the Fourier transform of a box with 3 × 3 × *N* unit cells qualitatively reproduces the diffuse pattern measured at *T* = 0.75 K (Fig. 3g). The good agreement is interpreted as evidence of prevalent *q* = (0, 0) correlations extending over three unit cells. They result from the local anisotropy and AF *J*_1_ and *J*_2_ couplings. We attempted to reproduce the pattern at *T* = 0.68 K by (i) considering parallel moment alignments on the *J*_3*b*_ bonds, (ii) populating only every third hexagon with a magnetic moment and suppressing the moment on other sides as shown in Fig. 3e, and (iii) limiting the size of coherent spin arrangements to 10 unit cells along the *a*- and *b*-axis. Figure 3h shows the Fourier transform of a box containing 10 × 10 x *N* unit cells, confirming the success of this approach. The model captures the main features of the measured *T* = 0.68 K pattern, *i*. *e*. the intensity at the main *hk* -positions and the smeared satellites at *q* = ($\frac{1}{3}$, 0) and its equivalents. At *T* = 0.25 K the ordering vector *q* = ($\frac{1}{3}$, 0, $\frac{1}{3}$) and its arms supersede any remains of the *q* = (0, 0) structure (see Fig. 3c). We can reproduce the diffraction pattern (see Fig. 3i) by Fourier transforming a 24 × 24 x *N* box containing three domains with the ($\frac{1}{3}$, 0),(0, $\frac{1}{3}$) and ($-\frac{1}{3},\,\frac{1}{3}$) propagation vectors and the amplitude modulated arrangement proposed in ref. ^23^. In this model *J*_3*a*_ and *J*_3*b*_ connect alternating parallel and antiparallel moment alignments, as shown in Fig. 3f. Our results suggest that the long-range magnetic order at low temperature is established as a compromise between the CEF anisotropy, AFM *J*_1_ and *J*_2_ couplings, and competing AFM *J*_3*a*_ and *J*_3*b*_ couplings.

### Itinerant electrons

The metallic nature of the *R*AgGe family adds complexity to understanding the magnetism. The itinerant electrons affect the degeneracy of frustrated states and can lead to significant further neighbour exchange couplings and Kondo screening. There exists a number of experimental evidences in YbAgGe, which do not comply with a localized moment picture. For instance, below T^*^ ≈ 20 K, the temperature at which the full *R*ln2 entropy is recovered^24,26^, our electric resistivity measurements with currents *I* parallel and perpendicular to the *c*-axis show deviations to the behavior at high temperature (see Fig. 1c). These data, together with previous magnetization and specific heat results reporting a stark deviation from the Curie-Weiss behavior below T^*^ and a Sommerfeld coefficient between 150 mJ/molK^2^ ≤ *γ* ≤ 1200 mJ/molK^2^ ^24,26^ indicate a link between conduction electrons and local magnetic degrees of freedom, suggesting hybridization of Yb 4*f* electrons with the conduction bands. Strong support for Kondo physics is also obtained from earlier neutron scattering results, reporting a substantial broadening of the quasielastic scattering and the first excited crystal-field level as a function of temperature^19,27^. Besides, our magnetization measured at high magnetic fields is incompatible with a localized picture (see Supplementary Information Note 4). Another argument for itineracy is the reduced and amplitude modulated ordered magnetic moment value as determined from neutron diffraction. Even inside the AFM LRO state at zero field, the ordered moment is only *μ*_*ord*_ ≈ 1.6*μ*_*B*_ on average^23^, implying a loss of ~60% when compared to the expected localized moment *μ*_*CEF*_ = 3.98*μ*_*B*_.

**Fig. 4:**
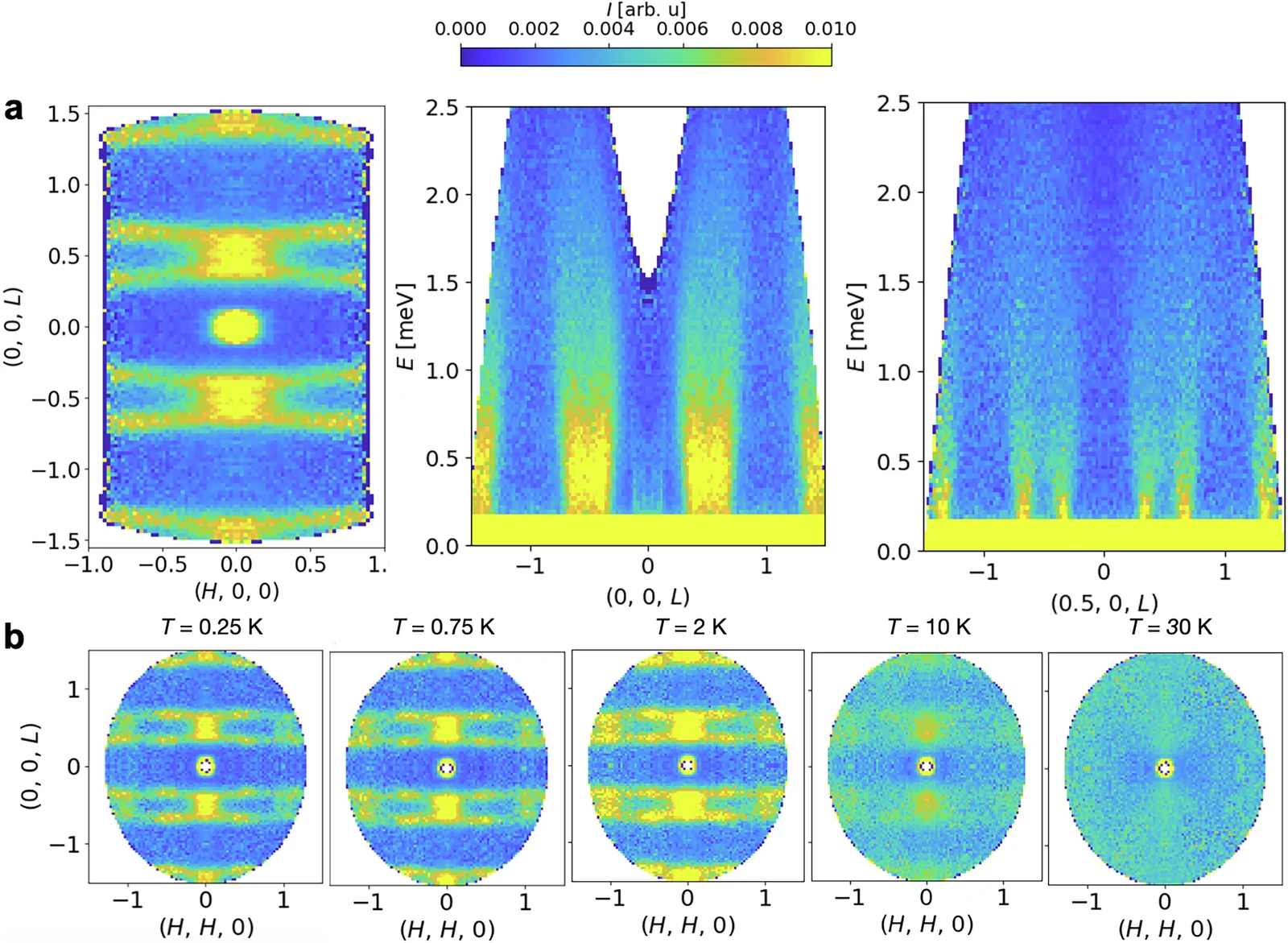
Dynamic properties of YbAgGe. **a** Magnetic excitation spectrum at *T* = 0.25 K and *μ*_0_*H* = 0 T in the (*H*, 0, *L*) plane at an energy transfer *E* = 0.3 meV, along (0, 0, *L*) and (1/2, 0, *L*). The data were measured on CNCS using an incident energy *E*_*i*_ = 3.32 meV and plotted with an energy integration *ΔE* = [0.2, 0.4] meV and reciprocal space integration *ΔQ* = [−0.1, 0.1] rlu in the first subpanel, and *ΔQ* = ±0.1 rlu perpendicular to the (0, 0, *L*) and (1/2, 0, *L*) directions. **b** Reciprocal space maps of the (*H*, *H*, *L*) plane at an energy transfer *E* = 0.3 meV at *T* = 0.25, 0.75, 2, 10 and 30 K. The data were measured with *E*_*i*_ = 3.32 meV using an energy integration range *ΔE* = [0.2, 0.4] meV and *ΔQ* = [−0.1, 0.1] rlu perpendicular to the (*H*, *H*, *L*) plane.

Our most striking counter to the localized picture is provided by our inelastic neutron scattering results. An analysis of the dipole-allowed transitions between the CEF ground state wavefunctions predicts the absence of observable magnetic excitations (see Supplementary Information Note 2 for details). This is in strong contrast to our experimental findings shown in Fig. 4a measured at *T* = 0.25 K and *μ*_0_*H* = 0 T on CNCS. We observe enhanced spectral weight along (*H*, 0, 1/3), (*H*, 0, 2/3) and around (0, 0, 1/2). The intensity is modulated mainly along the *c*-axis, but the spectrum also features an anisotropy in the Kagome plane. The excitations are column-like with decreasing intensity as function of energy transfer. No clear excitation gap is observed, instead we find that the excitation spectrum resembles an energy dependence reported previously for heavy-fermion metals^28–30^. In accordance to these results and an earlier report on YbAgGe^27^ we fitted the dynamic susceptibility with a quasielastic Lorentzian, and find that the function adequately describes our experimental results (see Supplementary Information Note 5 for details).

**Fig. 5:**
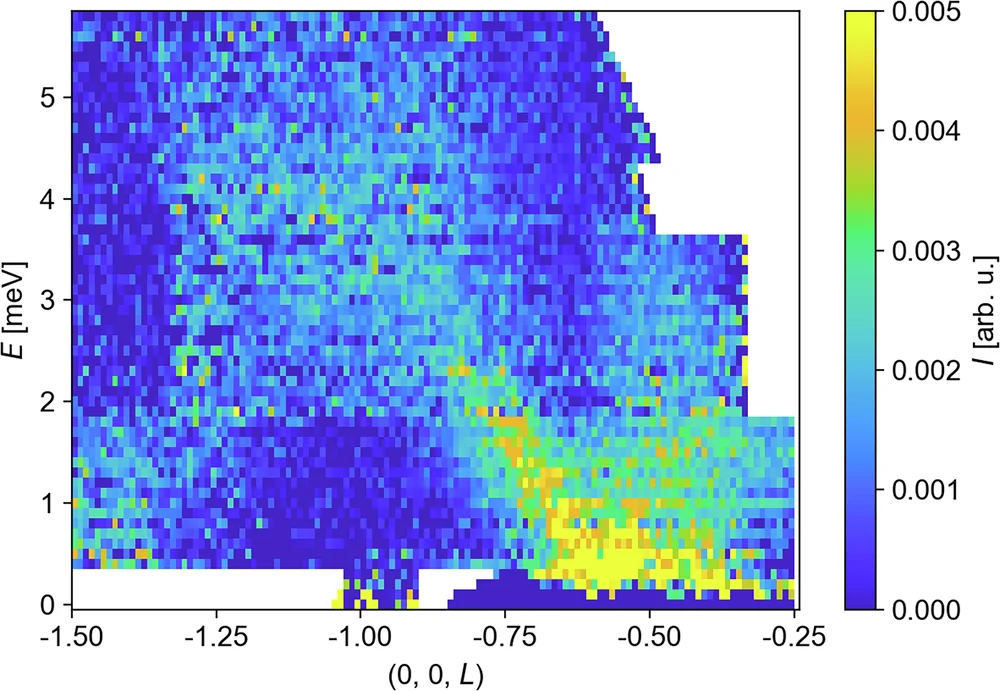
Dynamic properties in magnetic field. Magnetic excitation spectrum at *T* = 0.05 K and *μ*_0_*H* = 11 T along (0, 0, *L*). The background subtracted data were measured at CAMEA with a reciprocal space integration *ΔQ* = [−0.25, 0.25] rlu along the (*H*, *H*, 0) direction.

Remarkably, the magnetic excitation spectrum is not affected by moderate temperature changes. In Fig. 4b we display reciprocal space maps of the (*H*, *H*, *L*) plane for *E* = 0.3 meV measured at *T* = 0.25, 0.75, 2, 10, and 30 K. The results imply that magnetic fluctuations are unaffected by magnetic long-range order at *T*_*N*_ = 0.68 K. Notably, they are gradually weakened and suppressed only above T^*^ ≈ 20 K (see Supplementary Information Note 5 for details), matching the unusual temperature dependence of the electronic resistivity (see Fig. 1c). The INS spectra also reveal a nontrivial behaviour under magnetic field. Our results measured at CAMEA with *T* = 50 mK and *H*∥ [1, −1, 0] show that the magnetic fluctuations are only modified above *μ*_0_*H*  ≥  7 T where magnetic long-range order with ${q}_{z}\approx \frac{1}{3}$ is suppressed (see Supplementary Information Note 6 and ref. ^23^). In fact, at *μ*_0_*H* = 11 T we find that the excitations develop a dispersion along (0, 0, *L*) that is quasielastic at (0, 0, 1/3) and (0, 0, 2/3) and reaches a maximum at (0, 0, 1) with an energy transfer *E* ~ 3.5 meV (see Fig. 5). The full-width at half-maximum of the magnetic excitation on top of the dispersion is about three times larger than the instrumental resolution i.e. Δ*E* = 1.03(8) meV, providing evidence for a finite excitation lifetime.

We tested a variety of minimal local-moment Hamiltonians that retain ${q}_{z}\approx \frac{1}{3}$ to reproduce the magnetic excitation spectrum along (0, 0, *L*). Notably, we considered one-dimensional Heisenberg chains with an axial CEF anisotropy, anisotropic *xxz*-exchange interactions allowed by the crystal structure, and tested the effect of a magnetic field along *H*∥ [1, −1, 0] (see Supplementary Information Note 7 for details). As expected, we find that even a small anisotropy induces a finite gap in the excitation spectrum, contrasting the experimental observations at zero field and under magnetic field. This further supports that the microscopic nature of the magnetic properties in YbAgGe goes beyond the localized-moment picture, and indicates that magnetism is mediated by itinerant electrons.

## Discussion

YbAgGe is an intricate system showing elements of magnetic frustration, local moment and itinerant electrons physics. Our diffuse neutron scattering results, shown in Fig. 3a highlight that magnetic frustration stemming from the distorted Kagome plane and from orthogonal chain interactions plays an important role in the material. Localised exchange Hamiltonians are the standard approach for insulators and are also appropriate for metals possessing only a small density of states at the Fermi surface^31^. However, this approach is inappropriate for metals like YbAgGe, because the strong interactions between local magnetic moments and itinerant electrons trigger preeminent hybrid excitations, fainted by substantial damping effects^28,32–36^. One such example with qualitative similarities to YbAgGe is the frustrated Kagome lattice TbMn_6_Sn_6_^34–36^. The material features sharp acoustic magnons and overdamped flat-band excitations that induce in-plane structure factor patterns broadly resembling the diffuse scattering we observe in YbAgGe above *T*_*N*_ (see Fig. 3). The excitations in TbMn_6_Sn_6_ originate from flat-bands and chiral fluctuations localised on hexagonal plaquettes. However, since the ordered magnetic structure of YbAgGe is amplitude modulated and the moment directions are pinned by the CEF anisotropy, these short-range correlations are possibly governed by different fluctuations.

Most magnets with prominent itineracy require magnetic Hamiltonians which account for the electronic-band structure^37–39^. However, the small energy differences between different electronic configurations in Kondo metals still cause severe difficulties in theoretically predicting the electronic properties with sufficient accuracy, which complicates a mapping onto reliable effective Hamiltonians. Progress has been made in model cases of Kondo metals crystallizing in simple cubic lattice structures such as Ce*X*_3_ (*X* = Pd, In)^37–39^. Here, microscopic theoretical predictions have become possible, but their extension to complex structures such as non-centrosymmetric YbAgGe is not yet available. This step is required to quantitatively assess the effect of magnetic frustration in this material class. In fact, an intermediate route succeeding with this endeavor may include theoretical investigations of itinerant frustration hosting Kondo inactive 4*f*-ions. Examples include other materials in the *R*AgGe family, also revealing unusual frustrated quantum phases without the additional complexities imposed by the Kondo exchange interaction^22,40–42^. Such studies are important to deepen our understanding of how frustration is established in metallic hosts.

On a qualitative level, our various experimental observations can be grouped into four different temperature regions. Above *T* > T^*^ ≈ 20 K YbAgGe behaves like a paramagnetic intermetallic material. As the temperature is decreased below T^*^ a significant coupling between the itinerant electrons and the local magnetic moments is evidenced by the coincident change in the resistivity versus temperature behaviour and the emergence of spin-spin correlations in the INS data (see Figs. 1c and 4). The similarity to other Kondo metals^28–30,37^ suggests that the coupling in YbAgGe arises via Kondo screening leading to hybridized Yb-bands with coherent inter-band excitations. If so, the field dependence of the magnetic excitations further suggests that the hybridized Yb-bands are located in the eminent vicinity of the Fermi surface, because they are affected at moderate fields already. Confirmation of this hypothesis requires detailed knowledge of the Fermi surface topology, which can be gained using angle-resolved photoemission spectroscopy, scanning tunneling microscope spectroscopy, or De Haas-Van Alphen techniques. Unfortunately, technical challenges have restricted success of these methods in YbAgGe so far.

Below *T*_*SR*_ ≈ 2 K macroscopic heat capacity and electric resistivity measurements have shown that the magnetic properties change^24^. For 0.68 K < *T* < *T*_*SR*_ we find evidence for magnetic short-range correlations centered around *q* = (0, 0, 1/3), and long-range along the *c*-direction (see Figs. 2 and 3). The liquid-like state reveals similarities to a smectic phase with one long-range correlated axis only. While future theoretical efforts are required to elucidate the microscopic nature of this putative spin liquid-like phase, we find that it reveals growing correlations along the *c*-direction when the temperature is lowered, while correlations in the *ab*-plane remain short-ranged (see Fig. 2b, c). Using the localized Heisenberg picture, our analysis suggests AFM *J*_1_ and *J*_2_ couplings in the Kagome plane, and equal sized *J*_*c*1_ and *J*_*c*2_ interplane couplings to realize *q* = (0, 0, 1/3).

Below *T*_*N*_ = 0.68 K, magnetic long-range order is established along *q* = (1/3, 0, 1/3), in which in-plane ordering requires AFM *J*_*c*1_ and *J*_*c*2_ couplings and finite further neighbour terms in a localized picture (see Supplementary Information Note 3). The diffraction results^23^ report a significantly reduced magnetic moment of *μ*_*ord*_ ≈ 1.6*μ*_*B*_ with respect to the expected localized moment of *μ*_*CEF*_ = 3.98*μ*_*B*_ obtained from the CEF ground state wavefunction. These observations ascertain that a localized picture provides only a limited view, so that the itineracy plays an important role. This may be reflected also in the stability of ${q}_{z}\approx \frac{1}{3}$, which can be interpreted as robust Fermi surface nesting along the *c*-axis, resisting changes induced by an applied magnetic field. Along these lines the field modifications in *q*_*x*_ and *q*_*y*_ indicate almost degenerate nesting conditions in the hexagonal plane that can be tuned with magnetic field. The hypothesis is also supported by our inelastic neutron scattering results revealing an enhanced low-energy spectral weight along (*H*, *K*, 1/3). The enhanced spectral weight around (0, 0, 1/2) may further originate from competing nested Fermi surface sheets at larger energy transfers (see Supplementary Information Note 6 for details).

## Conclusion

The intricate interaction between the conduction electrons and localized moments in magnetic Kondo metals allows for the exploration of novel frustrated phases, whose interpretation remains challenging. In YbAgGe we observe enhanced dynamic fluctuations of 4*f* Yb-moments below T^*^ ≈ 20 K. Below *T*_*SR*_ ≈ 2 K the interaction between the itinerant electrons and local magnetic moments are modified, giving rise to a liquid-like state, which can be partially modeled via a localised Heisenberg Hamiltonian with AFM intraplane couplings. Magnetic long-range order is established below *T*_*N*_ = 0.68 K, suggesting AFM nearest-neighbour interplane couplings and finite further-neighbour terms. Altogether these experimental findings allude the following hierarchy of rather close energy scales ${E}_{CEF}^{splitting} > {E}_{KLH}^{exchange}\ge {E}_{frustrated}^{interactions}$. The case of YbAgGe testifies that effective model Hamiltonians that encode the electronic Fermi surface are required to accurately predict the microscopic properties of frustrated Kondo metals.

## Methods

### Crystal synthesis and macroscopic measurements

YbAgGe single crystals were grown from Ag- and Ge-rich high-temperature solutions and characterized as described in ref. ^26^. Sizable single crystals appropriate for inelastic neutron scattering were obtained through optimization of the synthesis process such as described in ref. ^23^. and^43^ (see Supplementary Information Note 1). Electric resistivity was measured down to *T* = 1.8 K using a Quantum Design PPMS-14 instrument. We used a standard AC four probe resistance technique (*f* = 16 Hz, *I* = 1 − 0.3 mA), where Pt leads were attached to a surface-polished YbAgGe single crystal using Epotek H2OE silver epoxy so that the current was flowing either along the crystallographic *c*-direction or within the *ab*-plane. The magnetization experiments in pulsed magnetic fields up to *μ*_0_*H* = 50 T were performed at the Dresden High Magnetic Field Laboratory. We used a compensated pickup-coil system in a pulse-field magnetometer with a home-built ^4^He cryostat and a pulsed magnet with an inner bore of 20 mm that was powered by a 1.44 MJ capacitor bank^44^.

### Crystal electric-field measurements

The crystal-electric field scheme of YbAgGe was probed at *T* = 3 K on a powdered sample enclosed in an Al can. The data were measured on the time-of-flight spectrometer MARI at ISIS Neutron and Muon Source, UK, using an incident neutron beam of *E*_*i*_ = 49.75 meV for which we found an elastic energy resolution of 3.98(2) meV full-width at half-maximum (FWHM), which is larger than the intrinsic resolution of the spectrometer. The spectrum with a wavevector transfer ∣*q*∣ < 4 Å^−1^ was fitted to two Gaussian peaks revealing CEF transitions centered at *E* = 12.0(4) meV. An additional peak emerges at *E* = 23(1) meV for 6 < ∣*q*∣ < 10 Å^−1^. The energy spectrum was analyzed using the PyCrystalField software package^21^. Further information can be found in Supplementary Information Note 2.

### Static and dynamic magnetism at zero magnetic field

The static and dynamic properties at *μ*_0_*H* = 0 T and *T* = 0.25−30 K were studied on the Cold Neutron Chopper Spectrometer (CNCS) at the Spallation Neutron Source (SNS), Oak Ridge National Laboratory, USA. A *m* ~ 2.5 g crystal oriented with (*H*, *H*, *L*) in the horizontal scattering plane was measured for 20 or 40 s per point over an angular range of 180 or 360^∘^ with a step size of 0.5^∘^. We used incident energies *E*_*i*_ = 12, 3.32, and 1.55 meV with chopper frequencies of 180 Hz on both double disk choppers, resulting in elastic energy resolutions Δ*E* = 1.2, 0.175, and 0.04 meV FWHM. The data was reduced with the Mantid software package^45^ and folded using the symmetry operations of $P\bar{6}2m$.

The magnetic field dependence of the dynamic properties was measured on the cold neutron multiplexing spectrometer CAMEA at the Paul Scherrer Institut, Switzerland^46^. The sample with *m* ~ 4 g consisted of two single crystals that were coaligned within 1^∘^ in the horizontal (*H*, *H*, *L*) plane. We used a vertical 11 T magnet with dilution insert, enabling measurements down to *T* = 50 mK. For *μ*_0_*H* = 0, 2, and 5.5 T, we used an incident energy *E*_*i*_ = 5 meV, providing an elastic line resolution of Δ*E* = 0.19 meV FWHM (see also Supplementary Information Note 6). At *μ*_0_*H* = 11 T, we used combination of different incident energies *E*_*i*_ = 3.6, 5, 6.8, 7.8, 8.7, and 9.6 meV with elastic line resolution of Δ*E* = 0.11 meV FWHM. The 60^∘^ wide detector was set at angles to cover a reciprocal space range of ~0.5 < ∣*q*∣ < ~2.3 Å^−1^ in the horizontal scattering plane optimized to follow the magnetic signal. We used a sample rotation step size of 0.5^∘^ to cover an angular range of 240^∘^ for detector settings ∣*q*∣ < ~1.5 Å^−1^, and 120^∘^ for ~0.8 < ∣*q*∣ < ~2.3 Å^−1^. About 25 h of statistics was collected at *μ*_0_*H* = 0, 2, and 5.5 T, and 71 h for *μ*_0_*H* = 11 T. The data was reduced with the MJOLNIR software package^47^. The background subtraction was performed via a powder average over the (*q*, *E*) range where no signal was present, similar to refs. ^48,49^.

## Supplementary information

Transparent Peer Review file

Supplementary Information

## Data Availability

The experimental data used in this work can be found using the link https://doi.org/10.5281/zenodo.19219126.

## Code availability

The code used to analyze the data presented in this study is available from the authors upon reasonable request.
