# Supplementary material for: Intricacies of frustrated magnetism in the Kondo metal YbAgGe
Source: Commun Mater. 2026 Jun 27;7(1):251. doi: 10.1038/s43246-026-01230-w (PMC13630540; doi:10.1038/s43246-026-01230-w)

nature portfolio

Peer Review File

Intricacies of Frustrated Magnetism in the Kondo Metal YbAgGe

Corresponding Author: Dr Daniel Mazzone

This file contains all reviewer reports in order by version, followed by all author rebuttals in order by version.

Version 0:

Reviewer comments:

Reviewer #1

(Remarks to the Author)
The manuscript presents interesting results on a distorted kagome metal.
I have a few minor comments.
- I suggest to include a short note on the low value of the residual resistivity ratio that may help the audience.
- A short note on the diffuse scattering in neutron experiments and its connection to the thermodynamic behavior, such as specific heat, may make it easier for the readers to follow.
- A brief explanation concerning the Kondo screening based on the experimental results on YbAgGe may be helpful for the reader.

Reviewer #2

(Remarks to the Author)
Referee report for
Intricacies of Frustrated Magnetism in the Kondo Metal YbAgGe. Mazzoni et al.

The paper poses a very interesting question, namely how does one consider frustration beyond the well considered localised electron case that is typically studied. The authors do this by studying, via single crystal inelastic and elastic neutron scattering, resistivity measurments and high field magnetisation measurements of a well known Kondo system: YbAgGe. The data that the authors present are very beautiful and novel and will be interesting to a wider field. However, in the current format, the analysis of the data and subsequent discussions are not sufficient to address the question adequately. In order to publish in nature communications the authors will need to focus their results more clearly. I therefore do not recommend publication of the paper in the current format. A significantly updated version can be considered for publication.

First, the authors do not highlight sufficiently the new results that they provide to the topic which are:

Determination of magnetic excitation patterns S(Q,ω) across various regions of the phase diagram.
Single crystal S(Q) for the various regions, q1 and q2, in the phase diagram with the determination of the magnetic wave vector in each region.
Simultaneous short and long range correlations similar to those observed in $SrEr_2O_4$ (Petrenko et al.)
Very close reproduction of S(Q), figure 3 of supplementary materials which should be a main result in the main paper.
A lack of a gap in the excitation pattern indicating itinerant electrons to the lowest temperatures. Again this discussion should be made stronger in the main paper.

In addition:
The paper claims to indicate a link between the itinerant nature of the sample and the local magnetic degree of freedom with, in particular, noting an anisotropy in the resistivity for different crystalline directions. I would argue that previous work (in particular the temperature dependence of the energy width of the QENS and CEF data by Matsumura et al.) are strong indicators of Kondo physics. However, the anisotropy of the resistivity is not in itself an indicator of Kondo physics since this may arise from an anisotropic crystal structure, as is the case here, anisotropic Fermi surface and band velocities, anisotropic scattering from defects or phonons, electronic nematic order or anisotropic magnetic fluctuations.

The CEF data provides no new information beyond that published by Matsumura et al. Do the authors observe a broadening of the CEF levels as observed by previous groups? Did the authors try to observe the CEF levels when measuring the single crystal on CNCS. It would be interesting to know whether the authors were able to check for any higher level CEF modes - that are possibly masked by the phonon excitations on CNCS (which can measure up to 80 meV).

The magnetic excitations are column like and are fitted using a QENS model with no gap. That is interesting however there is some inconsistency in the result: Supplementary Figure 7 (a): The linecut for q=(0 0 -1/2) could arguably show a gap at deltaE = 0.0125 meV while the equivalent data with fit in Figure 7(b) (bottom, T =0.25 K) does not. This is confusing. Typically if one wishes to show the absence of a gap from inelastic neutron scattering data then one would present a discussion on the energy resolution of the instrument and indicate the precision to which the absence of a gap is determined. This is missing.
Heisenberg/spin ice like exchange interaction scheme is able to reproduce the S(Q) maps rather well and the authors extract some information about the spin structure and the relevant exchange couplings. How do the authors calculate the S(Q) maps. Is it simply a spin structure with a unit spin vector? Why is the S(Q) calculation presented for T = 0.75 K performed for a 3x3xN box while T = 0.68 K for a 10x10xN box and for T = 0.25 K a 24x24xN box?

Why is there no mention of the T = 0.8 K phase transition, clearly observed in this work, in the early stages of the paper .

The authors then proceeded to employ LSWT to reproduce a pattern that does not match the data. LSWT is very sensitive to small variations - have the authors tried to optimise the spin-wave model parameters by comparing simulated neutron scattering data to experimental measurements using the sw_fitspec function?
Furthermore the anisotropic term is rather basic - why did you choose this term ? Are dipole exchange interactions relevant in this system? Why is there no discussion about spin-orbit coupling in this system which would lead to an anisotropic anisotropy more complex than presented in Eqn 1.

The authors mention a loss of moment, 1.6 μB in comparison to the expected 3.98 μB as determined by previous works. Are the authors able to extract the full moment from their INS results or is there still a discrepancy? What is the full moment determined from their INS work?

Line 226 - 230 in the main paper: there is a discussion about the excitations being overdamped. Please explain what you mean explicitly - overdamped with respect to what?
There is a discussion about the finite lifetime of the excitations - which is maybe an indication of quantum fluctuations and can be linked to the itinerant nature of the compound. However, again, in order to show that the data are lifetime limited, broadened in energy space, then a discussion about the energy resolution of the instrument is necessary. It should be noted again that Matsumura et al. has already observed broadening.

Finally, there are some linguistic errors that detract from the results.
Line 140: equidistant
Line 146 - do not start a paragraph with: In fact, or a reference to a previous paragraph.
Line 188: Linguistic issue.
Line 211 spelling error.
Line 301 non-magnetic magnetic Kondo …

Version 1:

Reviewer comments:

Reviewer #3

(Remarks to the Author)
Utilizing time-of-flight neutron spectroscopy, Mazzone et al. investigate the collective magnetism of the hexagonal intermetallic compound YbAgGe, where Yb^{3+} ions occupy distorted frustrated Kagome layers. The high-quality inelastic neutron scattering (INS) data presented in this work effectively captures the evolution of magnetism, from the paramagnetic diffuse scattering onsetting around 20 K to the complex spin dynamics observed within the magnetic ordered phase of YbAgGe arising below T_N= 0.68 K. While the high-temperature behavior can be qualitatively explained by a combination of strong crystal field anisotropy and competing exchange interactions, the low-temperature dynamics remains puzzling. Specifically, the presence of damped gapless columnar spin excitations at Q = (0,0,1/2) remains nearly insensitive to TN and cannot be modeled by a simple localized microscopic spin Hamiltonian. This observation, alongside the modulated magnetic order and thermodynamic data from heat capacity and resistivity, leads the authors to propose that itinerant effects promote Kondo coupling within this frustrated state.

Materials that intertwine magnetic frustration, itineracy, and Kondo physics are of significant interest to the hard condensed matter community. Furthermore, in my opinion, the authors have satisfactorily addressed the previous review comments. I then recommend this manuscript for publication in Communication Materials. I would, however, strongly suggest the authors address my only concern, which is related to the characterization of the crystal-field (CEF) Hamiltonian. To strengthen the work, the authors should include calculated INS spectra based on their CEF Hamiltonian for direct comparison with experimental data. Additionally, they should provide a comparison between the calculated single-ion magnetic susceptibility

derived from this Hamiltonian and the observed temperature dependence of the experimental magnetic susceptibility.

Version 2:

Reviewer comments:

Reviewer #3

(Remarks to the Author)
This referee thanks the authors for incorporating the suggested edits and further support this manuscript to be published in Communication Materials.

Open Access This Peer Review File is licensed under a Creative Commons Attribution 4.0 International License, which permits use, sharing, adaptation, distribution and reproduction in any medium or format, as long as you give appropriate credit to the original author(s) and the source, provide a link to the Creative Commons license, and indicate if changes were made.
In cases where reviewers are anonymous, credit should be given to 'Anonymous Referee' and the source.
The images or other third party material in this Peer Review File are included in the article's Creative Commons license, unless indicated otherwise in a credit line to the material. If material is not included in the article's Creative Commons license and your intended use is not permitted by statutory regulation or exceeds the permitted use, you will need to obtain permission directly from the copyright holder.
To view a copy of this license, visit https://creativecommons.org/licenses/by/4.0/

Point-by-point response to the reviewers' comments

Response to Reviewer #1

Reviewer:

The manuscript presents interesting results on a distorted kagome metal.

Our answer:

We appreciate that the referee finds our results interesting

Reviewer:

I suggest to include a short note on the low value of the residual resistivity ratio that may help the audience.

Our answer:

We thank the referee for this opportunity to clarify the origin of the low residual resistivity ratio in YbAgGe. An early report on the magnetic field dependence of the macroscopic properties (S. L. Bud'ko et al Phys Rev B 69, 014415 (2004)) has reported that the material shows a high residual resistivity value that can be efficiently suppressed with magnetic field. Notably, the resistivity is susceptible to the magnetic long-range order at low temperature, so that the residual resistivity drops by almost an order of magnitude when magnetic long-range order is suppressed with magnetic field. This provides evidence that the seemingly large residual resistivity value at zero field does not originate from crystalline disorder, but from magnetic scattering processes. In the revised caption of Figure 1c we now clarify the origin of the low residual resistivity ratio by writing: "Note that the material is known to possess a high residual resistivity value that does not arise from crystalline disorder, but stems from scattering processes which can be suppressed efficiently with magnetic field (S. L. Bud'ko et al Phys Rev B 69, 014415 (2004))"

Reviewer:

A short note on the diffuse scattering in neutron experiments and its connection to the thermodynamic behavior, such as specific heat, may make it easier for the readers to follow.

Our answer:

We agree with the referee that the connection between diffuse scattering observed in neutron experiments and its connection to the thermodynamic behavior can be strengthened. In the revised manuscript we now write in the last paragraph of page 2: "Previous thermodynamic measurements have shown that YbAgGe exhibits increased magnetic correlations already above T_N, for $T < 2$ K

(S. L. (Bud'ko et al Phys Rev B 69, 014415 (2004)). Here we reveal their microscopic origin via single crystal elastic scattering maps, which we measured on the Cold Neutron Chopper Spectrometer (CNCS) at ORNL. Figures 2a and 3a show elastic scattering maps in the (H, 0, L) and (H, K, -2/3) planes for T = 0.25, 0.68 and 0.75 K. Upon cooling below $T < 2$ K, diffuse scattering develops around q = (0, 0, 1/3) positions, which is sharp along the L direction but broad along H… "

Similarly in the second paragraph of page 7 we write: "Below $T_{SR} \approx 2$ K macroscopic heat capacity and electric resistivity measurement have shown that the magnetic properties change (Bud'ko et al Phys Rev B 69, 014415 (2004)). For $0.68 \text{ K} < T < T_{SR}$ we find evidence for magnetic short-range correlations centered around q = (0, 0, 1/3), and long-range along the c-direction (see Fig. 2)."

Reviewer:

A brief explanation concerning the Kondo screening based on the experimental results on YbAgGe may be helpful for the reader.

Our answer:

We thank the referee for this comment and have extended the text on page accordingly. Notably, we now write in the "itinerant electron section": " …We have collected a number of experimental evidences in YbAgGe, which do not comply with a localized moment picture. For instance, below $T^* \approx 20$ K, the temperature at which the full Rln2 entropy is recovered (Bud'ko et al Phys Rev B 69, 014415 (2004), E. Morosan et al JMMM 277, 298 (2004)), our electric resistivity measurements with currents I parallel and perpendicular to the c-axis show deviations to the behaviour at high temperature (see Fig. 1c). These data, together with previous magnetization and specific heat results reporting a stark deviation from the Curie-Weiss behavior below T* and a Sommerfeld coefficient between $150 \text{ mJ/molK}^2 \leq \gamma \leq 1200 \text{ mJ/molK}^2$ (Bud'ko et al Phys Rev B 69, 014415 (2004), E. Morosan et al JMMM 277, 298 (2004)) indicate a link between conduction electrons and local magnetic degrees of freedom, suggesting hybridization of Yb 4f electrons with the conduction bands. Strong support for Kondo physics is also obtained from earlier neutron scattering results, reporting a substantial broadening of the quasielastic scattering and the first excited crystal-field level as a function of temperature (T. Matsumura et al J. Phys. Soc. Jpn. 73, 2967 (2004), B Fak et al J. Phys: Cond Matt 17 301 (2004))"

Response to Reviewer #2

Reviewer:

The paper poses a very interesting question, namely how does one consider frustration beyond the well considered localised electron case that is typically studied. The authors do this by studying, via single crystal inelastic and elastic neutron scattering, resistivity measurments and high field magnetisation measurements of a well known Kondo system: YbAgGe. The data that the authors present are very beautiful and novel and will be interesting to a wider field. However, in the current format, the analysis of the data and subsequent discussions are not sufficient to address the question adequately. In order to publish in nature communications the authors will need to focus their results more clearly. I therefore do not recommend publication of the paper in the current format. A significantly updated version can be considered for publication.

Our answer:

We are pleased to read that the referee finds the question we address in our manuscript interesting and that they find our data "very beautiful", "novel" and of interest to a wider field. We acknowledge that the referee is of the opinion that the current form of the manuscript is not adequate for publication in *communication materials*. Following the referee's suggestion to focus the results in our manuscript more clearly, we resubmitted an updated version of the manuscript which we hope will merit the referee's recommendation for publication.

Reviewer:

First, the authors do not highlight sufficiently the new results that they provide to the topic which are: Determination of magnetic excitation patterns S(Q,ω) across various regions of the phase diagram. Single crystal S(Q) for the various regions, q1 and q2, in the phase diagram with the determination of the magnetic wave vector in each region. Simultaneous short and long range correlations similar to those observed in SrEr2O4 (Petrenko et al.) Very close reproduction of S(Q), figure 3 of supplementary materials which should be a main result in the main paper. A lack of a gap in the excitation pattern indicating itinerant electrons to the lowest temperatures. Again this discussion should be made stronger in the main paper.

Our answer:

We thank the referee for this constructive feedback. We agree that the previous version of our manuscript did indeed not sufficiently highlight our experimental neutron scattering observations. As a result we moved the information previously contained in SI Note 3 and 4 to the main text to deeper discuss the observed S(Q) pattern, the analysis of the correlation lengths and the simulation of the diffraction patterns (former Fig. S3 in the supplementary materials) showing close reproduction with the experimental S(Q). Following the suggestion of the referee we also strengthened the connection of the observed S(Q,w) neutron scattering pattern with itinerant magnetism. We agree with the referee that the column-like exaction, coupled with the lack of a gap

in the spectrum (despite CEF anisotropy) is a strong indication for itinerant magnetism. In the revised manuscript we now write in the second paragraph of page 5: “The excitations are column-like with decreasing intensity as function of energy transfer. No clear excitation gap was observed, , instead we find that the excitation spectrum resembles an energy dependence reported previously for heavy-fermion metals [J. Rossat-Mognid JMMM 76, 376 (1988), H, Kadowaki Phys. Rev. Lett. 92, 097204 (2004), W. Knafo Phys. Rev. B 70, 174401 (2004)]. In accordance to these results and an earlier report on YbAgGe (B Fak et al J. Phys: Cond Matt 17 301 (2004)) we fitted the dynamic susceptibility with a quasielastic Lorentzian, and find that the function adequately describes our experimental results (see SI Note 5 for details).“ and in the last paragraph before the discussion: “As expected, we find that even a small anisotropy induces a finite gap in the excitation spectrum, contrasting the experimental observations at zero field and under magnetic field. This further supports that the microscopic nature of the magnetic properties in YbAgGe go beyond the localized-moment picture, and indicate that magnetism is mediated by itinerant electrons“

Reviewer:

The paper claims to indicate a link between the itinerant nature of the sample and the local magnetic degree of freedom with, in particular, noting an anisotropy in the resistivity for different crystalline directions. I would argue that previous work (in particular the temperature dependence of the energy width of the QENS and CEF data by Matsumura et al.) are strong indicators of Kondo physics. However, the anisotropy of the resistivity is not in itself an indicator of Kondo physics since this may arise from an anisotropic crystal structure, as is the case here, anisotropic Fermi surface and band velocities, anisotropic scattering from defects or phonons, electronic nematic order or anisotropic magnetic fluctuations.

Our answer:

We thank the referee for this question. As written in the subsection “Itinerant electrons“, there are a number of experimental evidences that do not fit with a localized picture. Previous macroscopic measurements have found a stark deviation from the Curie-Weiss behavior below T* ≈ 20 K and a Sommerfeld coefficient between 150 mJ/molK2 and 1200 mJ/molK2 (Bud’ko et al Phys Rev B 69, 014415 (2004), E. Morosan et al JMMM 277, 298 (2004)). These two results are a first indication for Kondo physics. Our electric resistivity measurements match this temperature scale, because the deviations from a normal metallic behavior we observe also appear below T*. Furthermore, T* is also the temperature at which the full entropy is recovered (Bud’ko et al Phys Rev B 69, 014415 (2004), E. Morosan et al JMMM 277, 298 (2004)). To summarize these experimental results, and to clarify that this conclusion is drawn not only from the electric resistivity results we now write: “These data [meaning the electronic resistivity results] together with previous magnetization and specific heat results…, indicate a link between conduction electrons and local magnetic degrees of freedom, suggesting hybridization…“. Our inelastic neutron scatting experiment connects this

temperature scale to magnetic excitations, attributing them to Kondo-like behavior. We mention that in the caption of figure 2 in the revised manuscript we now also comment on the high residual resistivity value of the electric resistivity measurement, which does not arise from crystalline disorder in YbAgGe but can be reduced efficiently using a magnetic field.

We also found other experimental evidences that do not comply with a localized picture. Namely, the incompatibility of the high magnetization data with the crystal-field environment and the mismatch of the magnetic moment value in the long-range ordered state and the expected moment from the crystal-field scheme. The strongest evidence of itinerant magnetism is found in the low-energy excitation spectrum. The column like excitation and absence of a gap (despite anisotropy), the QENS temperature dependence discussed in SI Note 5 and B Fak et al J. Phys: Cond Matt 17 301 (2004)), coupled by the circumstance that the CEF scheme predicts the absence of observable excitations are strong arguments for itinerant magnetism. In the revised manuscript we now write on page 5 “The excitations are column-like with decreasing intensity as function of energy transfer. No clear excitation gap was observed, instead we find that the excitation spectrum resembles an energy dependence reported previously for heavy-fermion metals [J. Rossat-Mognid JMMM 76, 376 (1988), H, Kadowaki Phys. Rev. Lett. 92, 097204 (2004), W. Knafo Phys. Rev. B 70, 174401 (2004)]. In accordance to these results and an earlier report on YbAgGe (B Fak et al J. Phys: Cond Matt 17 301 (2004)) we fitted the dynamic susceptibility with a quasielastic Lorentzian, and find that the function adequately describes our experimental results (see SI Note 5 for details).“ and in the last paragraph before the discussion: “As expected, we find that even a small anisotropy induces a finite gap in the excitation spectrum, contrasting the experimental observations at zero field and under magnetic field. This further supports that the microscopic nature of the magnetic properties in YbAgGe go beyond the localized-moment picture, and indicates that magnetism is mediated by itinerant electrons“.

We agree with the referee that the previous neutron scattering studies of Matsumura et al and Fak et al strongly support the existence of Kondo physics in YbAgGe. In the revised manuscript we highlight their contribution in the first paragraph of the subsection “itinerant electrons“ via: “Strong support for Kondo physics is also obtained by earlier neutron scattering results, reporting a substantial broadening of the quasielastic scattering and the first excited crystal-field level as a function of temperature (T. Matsumura et al J. Phys. Soc. Jpn. 73, 2967 (2004), B Fak et al J. Phys: Cond Matt 17 301 (2004)).“

Reviewer:

The CEF data provides no new information beyond that published by Matsumura et al. Do the authors observe a broadening of the CEF levels as observed by previous groups? Did the authors try to observe the CEF levels when measuring the single crystal on CNCS. It would be interesting to

know whether the authors were able to check for any higher level CEF modes - that are possibly masked by the phonon excitations on CNCS (which can measure up to 80 meV).

Our answer:

We do not agree with the reviewers statement that our CEF provide no new information beyond that already published by Matsumura et al. Notably, we confirm the experimental results of Matsumura et al and their conclusions, but show a more complete dataset and report on further details revealed by our analysis. Firstly, while our and Matsumura et al's analysis report on the Steven's parameters of the analysed CEF data, we go in more detail in our supplementary Note 2, in which we report all eigenvectors and eigenvalues of the CEF scheme, report the expected moment value, and analyse the dipole-allowed transitions between the ground state wave function to give an estimation of the neutron cross-section expected in the ordered state. From this analysis we conclude that in a localized picture we would expect only very weak spin-wave excitations that we will not be able to observe (similar to what we have found in TmAgGe (Larsen et al Phys Rev B 107, 224419 (2023)). Moreover, the previous experiment from Matsumura et al reported inelastic spectra at two q-points only, whereas we show in Figure 1b the powder averaged spectra up to about 10 A-1, which provides new information. These data reveal phonon contributions and potentially phonon that admix with the first excited crystal-field level. If true, they may be related to the anomalies Nakanishi et al observed in their ultrasonic measurements (Y. Nakanishi et al J. Phys.: Conf. Ser. 273 012011 (2010)). However, we are of the opinion that such a discussion goes beyond the scope of this manuscript, and should be further investigated.

The CEF data we present were measured at T =3 K on the MARI spectrometer at the ISIS neutron and muon source using an incident energy of ~50 meV with an observed elastic line resolution of 4 meV. We have not investigated the temperature dependence of the CEF scheme as Matsumura did with better energy resolution. The width of the first excited CEF level matches the one Matsumura observed, but we cannot make a more detailed conclusions from the experimental data we have.

The largest incident energy we used on CNCS was Ei =12 meV. Thus, we did not investigate the crystal-field levels as function of temperature. Instead we focused on the low-energy excitations. However, we agree with the referee that a single-crystal investigation of the CEF levels would be of interest. The momentum dependence of the first CEF excitation may also give further information on the interaction parameters via a similar analysis to what we have employed in TmAgGe (Larsen et al Phys Rev B 107, 224419 (2023)). We hope that future studies will clarify these open questions.

Reviewer:

The magnetic excitations are column like and are fitted using a QENS model with no gap. That is interesting however there is some inconsistency in the result: Supplementary Figure 7 (a): The linecut for q=(0 0 -1/2) could arguably show a gap at deltaE = 0.0125 meV while the equivalent data with fit in Figure 7(b) (bottom, T =0.25 K) does not. This is confusing. Typically if one wishes

to show the absence of a gap from inelastic neutron scattering data then one would present a discussion on the energy resolution of the instrument and indicate the precision to which the absence of a gap is determined. This is missing.

Our answer:

Fits of column like excitations in heavy-fermion materials to quasielastic Lorentzians is a standard approach in the community. Because specific one-dimensional linecuts have been analysed previously in YbAgGe, we used the same formalism in Supplementary Note 5 (B Fak et al J. Phys: Cond Matt 17 301 (2004)). As written in the beginning of this note we used the three different incoming energies Ei =12, 3.32 and 1.55 meV at CNCS, resulting in elastic line resolutions of 1.2, 0.175 and 0.04 meV. The Figures 4b and 7a the reviewer is referring to were measured in the high resolution setup (resolution 0.04 meV), for which we only have base temperature data. The data in Fig. S7b are data stemming from an incident energy of 3.32 meV (resolution of 0.175 meV), with Fig. S7c being the quantitative analysis of these results. The resolution used for Fig. S7b is too broad to observe the putative small gap in Fig. S7a the referee is referring to. As such we do not feel that there is an inconsistency, because the resolution of the instrument is defined in the main text and we state in the caption which Ei was used for Fig. S7a and b.

Whether there is a gap at q = (0,0,1/2) with Ei =1.55 meV or not is a fair question. In our analysis the quasielastic fit reported in SI leads to a marginal better chi square when compared to regular Lorentzian or Gaussian line widths. Thus, we wrote in SI Note 5 "The high-resolution setting at Ei = 1.55 meV reveals no clear excitation gap at low energy transfer". As elaborated before column like excitations are often fitted to quasi elastic excitations in the heavy-fermion community, with a characteristic energy scale/width Γ. The temperature dependence of Γ is an indicator of critical slowing down in materials close to quantum criticality. Notably, if Γ approaches 0 (or goes beyond the elastic line resolution), it signals a divergence in imaginary time which is a requirement for quantum criticality. In YbAgGe Γ saturates at values above the elastic resolution of 0.175 meV below 5 K, suggesting that the excitations are not quantum critical. We comment on this behavior in the second paragraph of SI Note 5.

Reviewer:

Heisenberg/spin ice like exchange interaction scheme is able to reproduce the S(Q) maps rather well and the authors extract some information about the spin structure and the relevant exchange couplings. How do the authors calculate the S(Q) maps. Is it simply a spin structure with a unit spin vector? Why is the S(Q) calculation presented for T = 0.75 K performed for a 3x3xN box while T = 0.68 K for a 10x10xN box and for T = 0.25 K a 24x24xN box?

Our answer:

We used the Scatty program to calculate the S(Q) maps which is described in detail in J. A. M. Paddison et al Acta Cryst A75,14 (2019). Fast Fourier transform (FFT) is used alongside sampling theory to reduce high-frequency noise in the calculations of atomistic diffuse-scattering patterns.

We first created a box of (N1xN2xN3) Yb-atoms with a spin arrangement (each one discussed now in the main manuscript), which were then Fourier transformed and plotted using ppm files with the ParaView program for the Figures presented in Fig.3 g-i in the main manuscript. The majority of the boxes contained indeed unit or zero spin vectors, but in the case of amplitude modulated structure in Fig.3 i, the moments varied in length. We kept the number of unit cells along a and b equal N1=N2, but changed this number. For T = 0.75 K the number was small, N=3, as this limits the correlation length in accordance with our diffraction results and yields the broadening of the peaks in the simulation. Increasing this number increases the correlation length and subsequently sharpens the peaks. N=10 was sufficient for demonstration purposes of how the rings around the (h k 1/3) positions can be reproduced, while for the long-range pattern of Fig. 3i, a large N=24 was necessary to reproduce sharp Bragg peaks and collect adequate statistics over the three domains of the amplitude modulated structure.

We understand that this question is caused by insufficient explanations in the previous manuscript version and therefore extended the description of these simulations which is now in the main manuscript. In particular we write: "The diffuse scattering patterns correspond to spin-spin correlations that were modeled using fast Fourier transform and sampling theory to reduce the high-frequency noise in the calculations as implemented by the Scatty program (J. A. M. Paddison et al Acta Cryst A75,14 (2019))".

"Magnetic correlations confined to a few unit cells result in diffuse scattering around these hk positions, which is reproduced by a small number of unit cells along the a- and b-axes. Notably, he Fourier transform of a box with 3x3xN unit cells qualitatively reproduces the diffuse pattern measured at T = 0.72 K (Fig. 3g)." and

"We attempted to reproduce the pattern at T = 0.69 K by i) considering parallel moment alignments on the J3b bonds, ii) populating only every third hexagon with a magnetic moment and suppressing the moment on other sides as shown in Fig.3e, and iii) limiting the size of coherent spin arrangements to 10 unit cells along the a- and b-axis. "

Reviewer:

Why is there no mention of the T = 0.8 K phase transition, clearly observed in this work, in the early stages of the paper .

Our answer:

The zero-field behavior of YbAgGe can be roughly divided into four temperature regimes which is probably best seen in E. Morosan et al JMMM 277, 298 (2004). At high temperature T > T* ≈ 20 K

YbAgGe behaves as a parametric intermetallic material. Around T* the full entropy is recovered (see left panel of figure below). Below this temperature deviations from the Curie-Weiss behavior is observed, and it is also the temperature at which we start to see effects in the electrical resistivity and at which the low-energy excitations emerge (see Fig. 4c). T* has been attributed to the Kondo coherence temperature in the past. Below T < TSR ≈ 2 macroscopic heat capacity and electrical resistivity measurements (see right panel of figure below from (Morosan et al JMMM 277, 298 (2004) and Bud'ko et al Phys Rev B 69, 014415 (2004)) reveal an increase of magnetic correlations, which matches the emergence of the diffuse scattering we observe in the diffraction maps. The temperature dependence of the signatures observed in the macroscopic data is rather broad. Initially TSR was identified at 1 K, the temperature at which the resistivity deviates from the higher temperature behavior (Morosan et al JMMM 277, 298 (2004) and Bud'ko et al Phys Rev B 69, 014415 (2004)). In later publications the maximum of the heat capacity bump was used as the defining temperature for the q = (0,0,1/3) order which amounts to the 0.8 K phase transition the referee is referring to. In some cases such as in Y. Tokiwa et al Phys Rev Lett 111, 116401 (2013) only the transition into the long-range ordered state at TN = 0.68 K was defined (sharp transition in the heat capacity measurement).

We recognize that there is a confusion between the phase diagram we show in Supplementary Note 1 and the relation of the transition we indicate at 0.8 K with the short-range correlations observed below TSR = 2 K. We clarified this by adding the following sentence in the supplementary Note: "Macroscopic heat capacity and electrical resistivity measurements have found evidence for magnetic correlations below TSR ≈ 2 K at zero magnetic field (Morosan et al JMMM 277, 298 (2004) and Bud'ko et al Phys Rev B 69, 014415 (2004)). These correlations are signified by a broad response as a function of temperature which is maximal at 0.8 K, and has been used in the past to

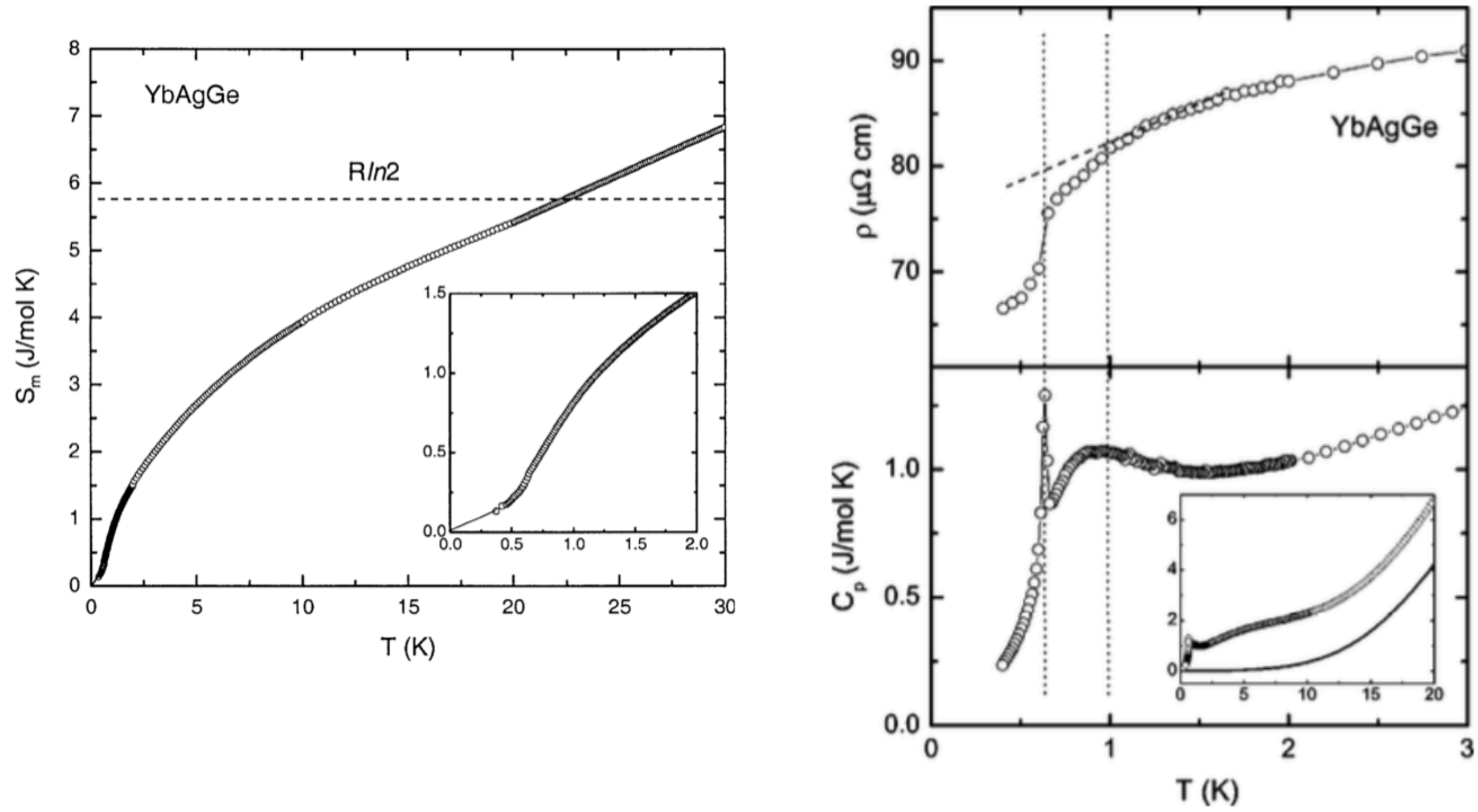

identify the phase boundary of these correlations in HT-phase diagrams. In the main manuscript we show that the temperature region TN < TSR is associated with short-range order with the propagation vector (0,0,1/3) that is correlated mainly along the c-axis.“

Reviewer:

The authors then proceeded to employ LSWT to reproduce a pattern that does not match the data. LSWT is very sensitive to small variations - have the authors tried to optimise the spin-wave model parameters by comparing simulated neutron scattering data to experimental measurements using the sw_fitspec function?
Furthermore the anisotropic term is rather basic - why did you choose this term ? Are dipole exchange interactions relevant in this system? Why is there no discussion about spin-orbit coupling in this system which would lead to an anisotropic anisotropy more complex than presented in Eqn 1.

Our answer:

We thank the reviewer for this question. We first recall that the CEF ground state wave function predicts non-observable spin waves in YbAgGe (see SI Note 2). However, in our experiments we observe column-like excitations that can be fitted with quasielastic Lorentzians that are often used in heavy-fermion systems. These excitations are not affected by the emergence of magnetic long-range order at 0.68 K, but are rather connected to the temperature scale at T* ≈ 20 K which according to previous experiments is likely governed by the Kondo interaction (see for instance Morosan et al JMMM 277, 298 (2004) and Bud’ko et al Phys Rev B 69, 014415 (2004)). These results suggest that the low-energy excitations we observe arise from itinerant electrons rather than from localized magnetism.
The quasielastic nature of the excitations are particularly contrasting an interpretation towards a localized picture, because YbAgGe features an appreciable CEF anisotropy between the Kagome plane and the c-axis. Thus, one would naively expect a gapped excitation spectrum. This statement is further strengthened if we consider the magnetic field dependence of the excitations. Naively, we would expect a Zeeman splitting of the magnon bands if the excitations arose from localized moments. In our case however, a magnetic field applied within the Kagome plane has no impact on the magnetic excitation spectrum up to ~7 T, and is thus also not susceptible to the various long-range ordered states occurring in the HT-phase diagram (see SI Figure 1). At 11 T we find that the expectation spectrum develops a dispersion, but even at this field the spectrum remains ungapped. As elaborated in the discussion part we interpret these excitations as inter-band excitations arising from the Yb-bands that are located in the imminent vicinity of the Fermi surface.
Nevertheless, we attempted to test if minimal localized models succeed in reproducing the observed excitation spectrum. Notably, we showed that in local models a small anisotropy is sufficient to induce a gap in the excitation spectrum which is inconsistent with our observations. We agree with the referee that our models may be basic, but they are rooted in justifiable assumptions. Firstly, we

have observed that the correlation lengths are much larger along the crystalline c-axis than in the Kagome plane, and that the qz=1/3 component of the propagation vector is robust as a function of temperature and field (which in fact may result from robust Fermi surface nesting along the c-axis). Thus, a simplistic model considering independent tubes along the c-axis retaining a qz propagation vector is a reasonable model based on these experimental observation. Such a model is realized in one-dimensional Heisenberg chains with two antiferromagnetic interactions Jc1 and Jc2 at ~0.4 ratio. The values were adjusted such that they very broadly cover the excitation range observed at 11 T. We then introduced a very small anisotropy to mimic the axial crystal-field anisotropy to force the spins into the Kagome plane (as reported by diffraction, seeLarsen et al Phys. Rev. B 104, 045524 (2021)). As shown in SI Note 7 a very small anisotropy is sufficient to induce a gap in the excitations spectrum. A similar behavior is found if a magnetic field is applied (see supplementary figure 7). We then decided to model the crystal-field anisotropy via anisotropic exchanges. The reason for this model is also motivated to explore a case of anisotropic interactions, which metallic systems are prone to. Similarly, we find that a small anisotropy induces a gap. In the revised supplementary Note 7 we clarified our reasoning by writing: “We attempted to model the magnetic excitation spectrum of YbAgGe using a minimal local-moment Hamiltonian. The diffraction experiments in Fig.2 and 3 of the main manuscript reveal the correlations length along the c-axis are largely exceeding the ones in the Kagome plane. The excitation spectrum shows an energy dependence of the fluctuations that takes place mainly along the L-direction, for which earlier diffraction results reveal a robust qz ≈ 1/3 component of the magnetic wavevector (see also SI Note1) (Larsen et al Phys. Rev. B 104, 045524 (2021)). Thus, we first looked into one-dimensional Heisenberg chain Hamiltonians with interaction parameters that retain the qz ≈ 1/3 periodicity and spin-wave boundaries that broadly reflect the excitation energy range of the u0H = 11 T spectrum shown in Fig. 5 of the main manuscript“ and “…Both, the finite axial anisotropy A<0 mimicking the crystal field anisotropy and non-zero magnetic field lead to a gaped excitation spectrum, as shown in Figures S7 c and d…“

We agree with the referee that models with reasonable behavior should be fitted to the experimental data with programs like SpinW to optimize the parameters and to obtain robust results. However, the fact is that we did not find a physically justifiable minimal local model that can be fitted to the experimental data. We argue that this is rooted in the nature of these excitations and that they do not arise from local interactions but require theoretical approaches that include the electronic degree of freedom. We also agree that our anisotropy term is rather basic and chosen to simulate the axial anisotropy that originates from the CEF environment. However, despite the basic anisotropy our finding is robust in the sense that a small anisotropy leads to a gapped spectrum which is contrasting our experimental observation. This is similarly valid for spin-orbit coupling terms.

The dipole-dipole term can be approximated by roughly J ~ 5/2\mu_0/4\pi\mu^2/r3 with mu_0 being the vacuum permeability, \mu the ordered moment and r the distance between two magnetic ions. The nearest neighbor ion distance is 3.7A and the average ordered moment is 1.6 muB, which yields J ~ 33 mK which is about a factor 20 times smaller than the ordering temperature. Thus, we are confident that dipole-dipole effects do not play an essential role and can be neglected in YbAgGe.

Reviewer:

The authors mention a loss of moment, 1.6 μB in comparison to the expected 3.98 μB as determined by previous works. Are the authors able to extract the full moment from their INS results or is there still a discrepancy? What is the full moment determined from their INS work?

Our answer:

We thank the referee for this excellent question. Indeed it would be favorable to quantitatively assess the elastic and inelastic scattering contribution in our study on CNCS via the total moment sum rule, similar to what has been done for MnSb2O6 in E. Chan et al Phys. Rev. B 107, 144420 (2023). We are afraid that such an analysis is unfortunately not possible in our case. The ideal dataset to perform such an analysis are the measurements we have performed with Ei = 12 meV to cover almost all spectral weight up to the first excited crystal-field level. However, since at this Ei the spectra are somewhat polluted with additional scattering and aluminum lines (see Fig. 2a of the main manuscript and Fig. below) a proper background subtraction is required to perform a meaningful analysis. Unfortunately the highest temperature measured with this Ei was 15 K, at which appreciable magnetic scattering is still present in the inelastic channel (see Figure below). With the Ei = 3.32 meV dataset on the other hand we could only reach reliable energy transfers up to 2.7 meV, above which a substantial fraction of inelastic spectral weight is still present (see Fig. 4a, Supplementary Fig. 5 b and B Fak et al J. Phys: Cond Matt 17 301 (2004)). Therefore, we unfortunately miss the required data to

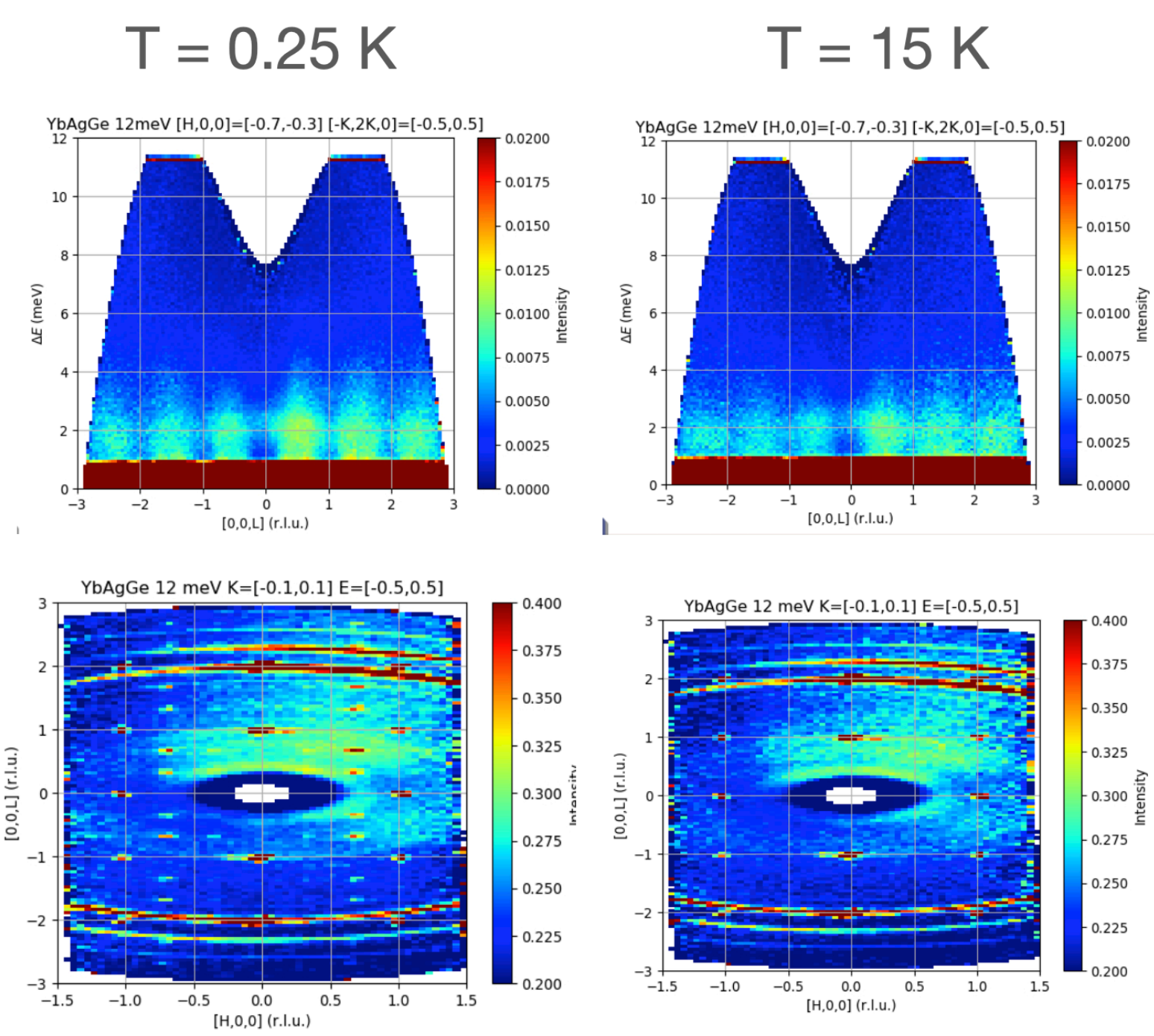

perform a meaningful analysis of the total moment. Unfortunate circumstances during our CNCS experiment (performed during Covid) also drastically complicate a transformation of the scattering intensity into absolute units, without imposing systematic errors. Thus, while we find the referee's input excellent, we are afraid that the present work cannot accommodate the request and have to leave such an analysis for future studies.

Reviewer:

Line 226 - 230 in the main paper: there is a discussion about the excitations being overdamped. Please explain what you mean explicitly - overdamped with respect to what?

Our answer:

We thank the reviewer for this comment. The term "overdamped" was a bad choice of word. We intended to write that the excitations can be fitted well with a quasielastic Lorentzian. We modified the wording. The section now reads: "The excitations are column-like with decreasing intensity as function of energy transfer. No clear excitation gap is observed, instead we find that the excitation spectrum resembles an energy dependence reported previously for heavy-fermion metals [J. Rossat-Mignod JMMM 76, 376 (1988), H, Kadowaki Phys. Rev. Lett. 92, 097204 (2004), W. Knafo Phys. Rev. B 70, 174401 (2004)]. In accordance to these results and an earlier report on YbAgGe (B Fak et al J. Phys: Cond Matt 17 301 (2004)) we fitted the dynamic susceptibility with a quasielastic Lorentzian, and find that the function adequately describes our experimental results (see SI Note 5 for details)."

Reviewer:

There is a discussion about the finite lifetime of the excitations - which is maybe an indication of quantum fluctuations and can be linked to the itinerant nature of the compound. However, again, in order to show that the data are lifetime limited, broadened in energy space, then a discussion about the energy resolution of the instrument is necessary. It should be noted again that Matsumura et al. has already observed broadening.

Our answer:

We thank the referee for this comment. We agree that Matsumura et al (and also Fak et al) have already observed a broadening of the elastic line and the CEF excitations. Whereas Fak et al fitted the elastic line to a quasielastic Lorentzian in accordance to other heavy-fermion compounds (see previous point). Matsumura used a Lorentzian function to describe the broadening of the first crystal-field level. As described in the third answer to the referee, in the revised manuscript we have made Matsumura's result more apparent by writing: in the "itinerant" electron section: "Strong support for Kondo physics is also obtained by earlier neutron scattering results, reporting a substantial broadening of the quasielastic scattering and the first excited crystal field level as a

function of temperature (T. Matsumura et al J. Phys. Soc. Jpn. 73, 2967 (2004), B Fak et al J. Phys: Cond Matt 17 301 (2004)).“

However, none of these studies have explored the magnetic field dependence of the low-energy fluctuations, for which we find that they are unaffected up to ~7T despite crossing several magnetic phase transitions. In fact, we find that a dispersion is developed only at 11 T (the maximum field we could reach), but the excitation remains quasielastic and broad. We are of the opinion that this result is new and goes beyond the results reported in Matsumura's paper.

Regarding the energy resolution of the instrument mentioned by the referee, we note that we have reported the elastic energy resolution of the instrument in Method section, but agree with the referee that we should also have indicated the instrument resolution at an energy transfer of 3.5 meV which is ≈300 uev, about 3 times sharper than the dispersion width of ~1 meV we observed.

In the revised manuscript we now write: “The full-width at half-maximum of the magnetic excitation on top of the dispersion is about three times larger than the instrumental resolution, i.e. \Delta E = 1.03(8) meV, providing evidence for a finite excitation lifetime. “

Reviewer:

Finally, there are some linguistic errors that detract from the results.
Line 140: equidistant
Line 146 - do not start a paragraph with: In fact, or a reference to a previous paragraph.
Line 188: Linguistic issue.
Line 211 spelling error.
Line 301 non-magnetic magnetic Kondo …

Our answer:

We thank the reviewer for raising awareness for these linguistic errors. They have been corrected in the revised manuscript.

Point-by-point response to the reviewers' comments

Response to Reviewer #3

Reviewer:

Materials that intertwine magnetic frustration, itineracy, and Kondo physics are of significant interest to the hard condensed matter community. Furthermore, in my opinion, the authors have satisfactorily addressed the previous review comments. I then recommend this manuscript for publication in Communication Materials.

Our answer:

We appreciate that the referee finds our research work of importance, their positive assessment of the quality of our resubmission and the recommendation for publication in Communication Materials.

Reviewer:

To strengthen the work, the authors should include calculated INS spectra based on their CEF Hamiltonian for direct comparison with experimental data. Additionally, they should provide a comparison between the calculated single-ion magnetic susceptibility derived from this Hamiltonian and the observed temperature dependence of the experimental magnetic susceptibility.

Our answer:

We thank the referee for this constructive criticism. In accordance to their suggestion we have significantly expanded our Supplementary Note 2, which reports on the details of the crystal-electric field refinement. Notably, we have added a new Supplementary Figure 2, showing in panel (a) the experimental neutron spectrum for $|q| < 4$ A^{-1} together with the predicted spectrum obtained from the refined crystal-electric field (CEF) parameters. We also report on details on how we modeled the experimental background. The referee's suggestion also gave us the opportunity to report on the quality of the fit, and to deepen our discussion on why we concluded that the non dispersing excitations at larger energy transfers are of phononic origin. In panel (b) we show experimental spectra for $|q| < 4$ A^{-1} together with $6 < |q| < 10$ A^{-1} and also performed a fit to the data when the excitations at $E = 23$ and 36 meV are included in the refinement. The significantly worse quality of fit, destabilized refinement of the 12 meV excitation and |q|-dependence of the experimentally determined excitation spectrum suggests a phononic nature of the E = 23 and 36 meV transitions.

The predicted temperature dependence of the magnetic susceptibility is shown in panel (c) of Suppl. Fig. 2. We note that it reveals a similar behavior to the one reported in Matsumura et al J. Phys. Soc. Jpn. 73, 2967 (2004). In their Figure 3 (see also below), the authors compare the calculated

susceptibility to experimental results. Deviations from the experimental results have been attributed to itinerant effects and large fluctuations along the c-axis. We find this assessment sound and elaborate in our manuscript that both effects are significant in YbAgGe. We note that we decided to directly refer to the paper of Matsumura et al rather than showing experimental data in Supplementary Note 2, because we have not measured any temperature dependent susceptibility data for our work. We also mention that we have reanalyzed the CEF spectra in view of this review process and further optimized the B^0_2 parameter, which leaded to very minor modifications of the reported eigenvectors and eigenvalues.

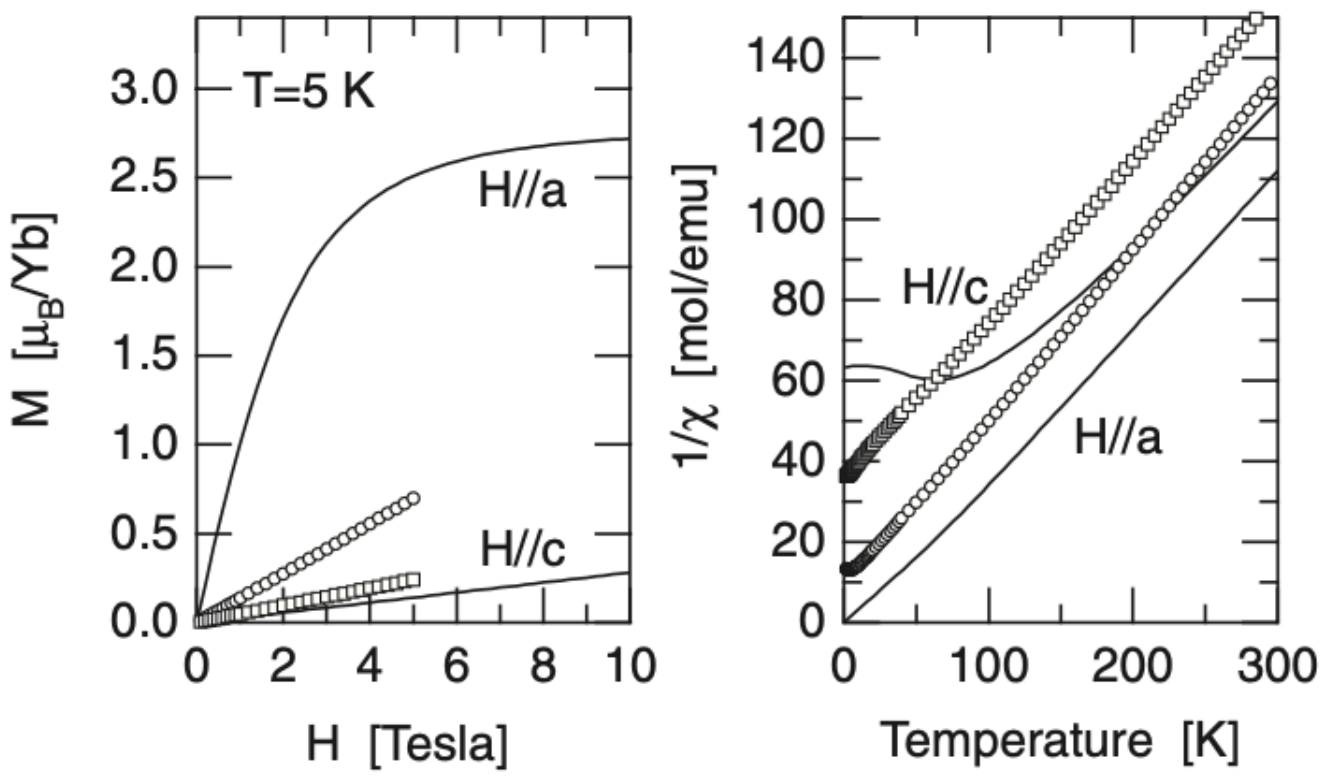

Supplement: Supplementary file 1 [file 43246_2026_1230_MOESM1_ESM.pdf]
