# Supplementary material for: Intricacies of frustrated magnetism in the Kondo metal YbAgGe
Source: Commun Mater. 2026 Jun 27;7(1):251. doi: 10.1038/s43246-026-01230-w (PMC13630540; doi:10.1038/s43246-026-01230-w)
Supplement: Supplementary file 2 [file 43246_2026_1230_MOESM2_ESM.pdf]

Supplementary information: Intricacies of Frustrated Magnetism in the Kondo Metal YbAgGe

D. G. Mazzone,[1,*] C. B. Larsen,[1] B. Ueland,[2,3] X. Boraley,[1] D. M. Pajerowski,[4] Y. Skourski,[5] J. Taylor,[6,4] B. Fåk,[7] S. L. Bud'ko,[2,3] R. McQueeney,[2,3] P. C. Canfield,[2,3] and O. Zaharko[1,†]

[1]PSI Center for Neutron and Muon Sciences, 5232 Villigen PSI, Switzerland
[2]Department of Physics and Astronomy, Iowa State University, Ames, Iowa 50011, USA
[3]Ames National Laboratory, Iowa State University, Ames, Iowa 50011, USA
[4]Neutron Scattering Division, Oak Ridge National Laboratory, Oak Ridge, Tennessee 37831, USA
[5]Dresden High Magnetic Field Laboratory (HLD-EMFL), Helmholtz-Zentrum Dresden-Rossendorf, 01328 Dresden, Germany
[6]ISIS Facility, STFC Rutherford Appleton Laboratory, Harwell Science and Innovation Campus, Oxfordshire OX11 0QX, United Kingdom
[7]Institut Laue-Langevin, 71 Avenue des Martyrs, CS20156, 38042 Grenoble Cédex 9, France

Supplementary Note 1: Single crystal growth and magnetic phase diagram

YbAgGe single crystals were grown from a Ag- and Ge-rich high-temperature ternary solution[1] and characterized based on the procedure described in Ref.[2]. In order to grow a significant number of large single crystals of YbAgGe, the growth was optimized in several ways. Roughly 10 g of high purity Yb, Ag, and Ge were placed in a 5 ml Al_2O_3 crucible in a molar ratio of Yb0:14Ag0:645Ge0:215. The Yb with 99.99+% purity was obtained from the Ames Laboratory Materials Preparation Center. A second 5 ml Al_2O_3 crucible, filled with silica wool, was placed on top of the growth crucible to act as a filter and catch for excess liquid[2]. The growth and catch crucibles were sealed into an evacuated amorphous silica tube and placed in a resistively heated box furnace. In order to partially control nucleation, the growth ampoules were placed close to the inner edges of the furnace door. We found that larger crystals often grew on the side of the crucible facing this colder spot in the furnace. The furnace was heated to 1100 °C over 4 hours, and heated further to 1190 °C over an additional hour. The furnace was then cooled to 1090 °C over 5 hours and then slow cooled to 840 °C over 200 hours. The growth ampoules were removed from the furnace at 840 °C and placed in a centrifuge[1] for separation of the excess liquid from the YbAgGe single crystals. Depending on nucleation, single crystals as large as 1 - 2 grams could be obtained (see inset of Supplementary (Suppl.) Fig. 1).

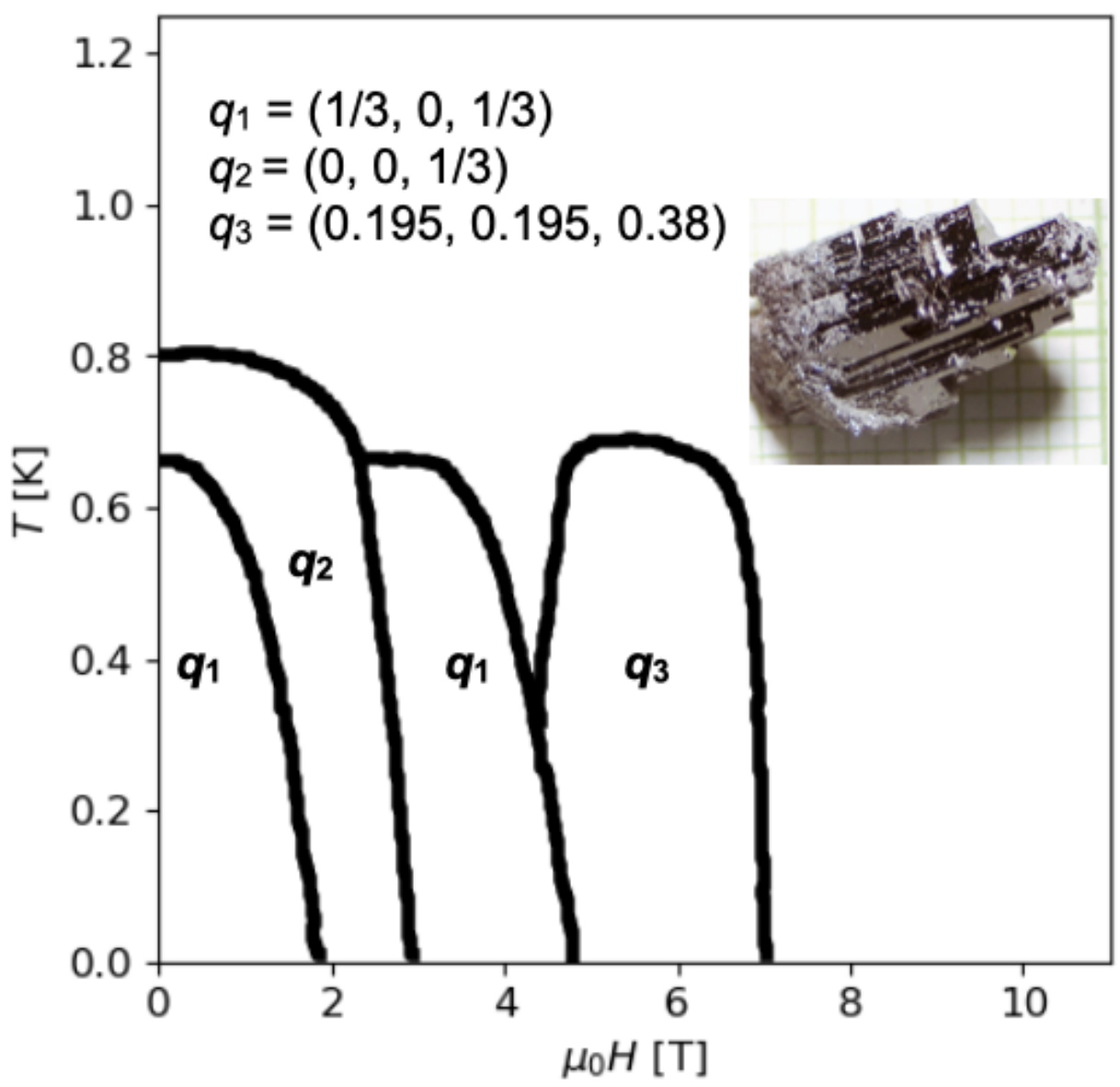

Supplementary Figure 1: **Schematic HT-phase diagram for $H||[1,-1,0]$.** Schematic magnetic phase diagram of magnetic long-range order in YbAgGe for $H||[1,-1,0]$ with corresponding magnetic wavevectors q_{1-3}. The diagram has been reproduced from Ref.[3]. Inset: Picture of a YbAgGe single crystal.

Macroscopic heat capacity and electrical resistivity measurements have found evidence for magnetic correlations below $T_{SR} \approx 2$ K at zero magnetic field[2,4]. These correlations are signified by a broad response as a function of temperature which is maximal at 0.8 K, and has been used in the past to identify the phase boundary of these correlations in HT-phase diagrams. In the main manuscript we show that the temperature region $T_N < T_{SR}$ is associated with short-range order with the propagation vector (0, 0, $\frac{1}{3}$) that is correlated mainly along the c-axis. Antiferromagnetic (AFM) long-range order in YbAgGe is established below $T_N = 0.68$ K with a propagation vector $q = (\frac{1}{3}, 0, \frac{1}{3})$[3]. A magnetic field applied in the Kagome plane triggers a series of metamagnetic transitions to other magnetic states with different periodicity as shown in Suppl. Fig. 1. Above $\mu_0 H \approx 7$ T ($H \parallel$ [1, -1, 0]) these modulations vanish, so that eventually only an induced ferromagnetic

Supplementary Table I: Eigenvectors and eigenvalues of the CEF scheme of the Yb^{3+} ion in YbAgGe.

E (meV)	$\vert-\frac{7}{2}\rangle$	$\vert-\frac{5}{2}\rangle$	$\vert-\frac{3}{2}\rangle$	$\vert-\frac{1}{2}\rangle$	$\vert\frac{1}{2}\rangle$	$\vert\frac{3}{2}\rangle$	$\vert\frac{5}{2}\rangle$	$\vert\frac{7}{2}\rangle$
0.000	-0.189	0.0	-0.578	0.0	-0.695	0.0	-0.385	0.0
0.000	0.0	-0.385	0.0	-0.695	0.0	-0.578	0.0	-0.189
12.165	-0.535	0.0	-0.521	0.0	0.234	0.0	0.622	0.0
12.165	0.0	-0.622	0.0	-0.234	0.0	0.521	0.0	0.535
19.864	0.0	0.533	0.0	-0.281	0.0	-0.263	0.0	0.753
19.864	-0.753	0.0	0.263	0.0	0.281	0.0	-0.533	0.0
27.805	0.333	0.0	-0.57	0.0	0.619	0.0	-0.424	0.0
27.805	0.0	0.424	0.0	-0.619	0.0	0.57	0.0	-0.333

(FM) component remains. We mention that previous macroscopic studies revealed additional correlated signatures in the HT-phase diagram of YbAgGe[5].

Supplementary Note 2: Crystal electric-field refinement

The energy spectrum of a polycrystalline YbAgGe sample measured on the MARI spectrometer at ISIS with $E_i = 49.75$ meV and $T = 3$ K revealed nondispersing excitations at $E = 12.0(4)$, 23(1) and 36(1) meV. The first feature reveals intensity prevailing below the wavevector transfers $|q| < 4$ Å^{-1}, which we attributed to a crystal electric-field (CEF) transition. Fits of this transition against the CEF Hamiltonian $H_{CEF} = \sum_{n,m} B_n^m O_n^m$, with O_n^m being Stevens operators and B_n^m CEF parameters by PyCrystalField python package[6] using the z-direction as the local quantization axis reveal $B_2^0 \approx 0.137$ meV, $B_2^2 \approx$ -1.27 meV ($\chi^2 = 0.17$) and negligible higher order CEF parameters. The best fit to the data and resulting set of eignevectors and eigenvalues are shown in Suppl. Fig. 2a and Tabl. I, respectively, establishing a ground state doublet $|\Psi^{\pm}\rangle$ = -0.189$|\mp7/2\rangle$ - 0.385$|\pm5/2\rangle$ - 0.578$|\mp3/2\rangle$ - 0.695$|\pm1/2\rangle$ with a localized moment of $\mu_{CEF} = 3.98\mu_B$ and significant axial anisotropy, g-tensor = (7.958, 0.0247, 0.0303). Attempts to include the second and third peaks with intensity dominating at $6 < |q| < 10$ Å^{-1} destabilized the refinement and worsens the fit of the $E = 12.0(4)$ meV transition. Along with the $|q|$ dependence of the excitation spectrum this suggests a phononic origin of the $E = 23(1)$ and 36(1) meV excitations (see Suppl. Fig. 2b). The calculated inverse susceptibility χ^{-1} and magnetization M are shown in Suppl. Figs. 2c and 4, respectively. In agreement with previous reports[7] the predicted macroscopic quantities reveal some deviations from the experimental results (see Ref.[7]), which have been attributed to itinerant effects in the material and large fluctuations along the c-axis.

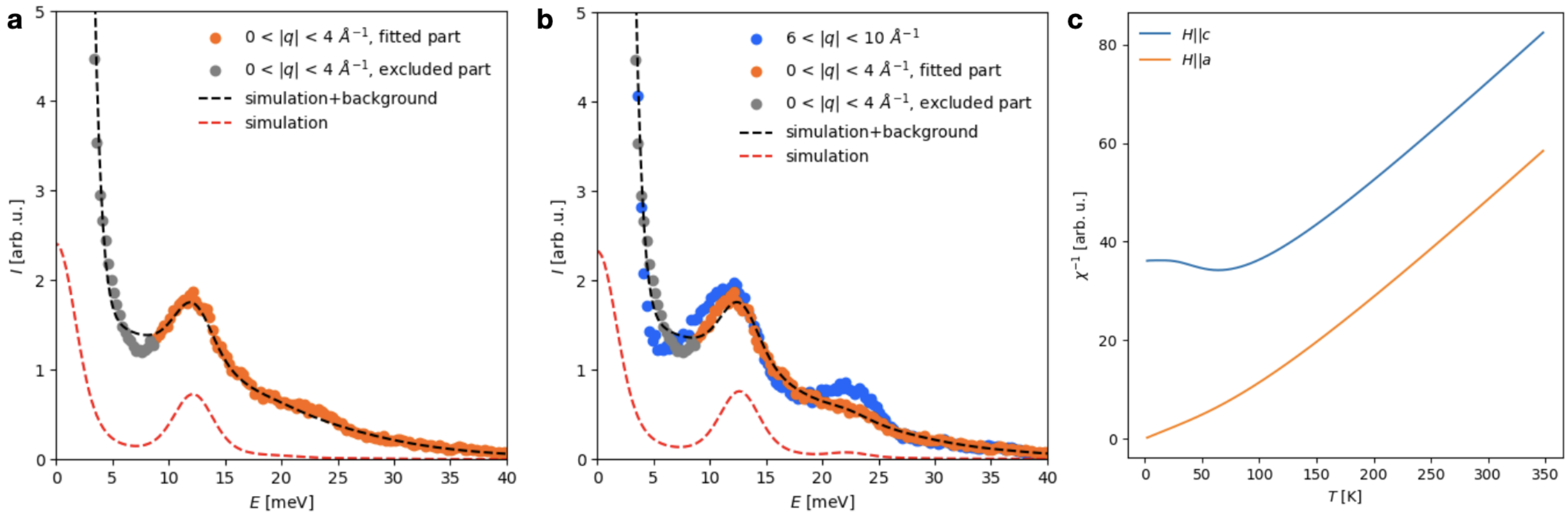

Supplementary Figure 2: **Crystal electric-field refinement. a** Neutron scattering spectrum obtained on MARI integrated over a wavevector transfer $|q| < 4$ Å^{-1} in gray and orange. The orange data points were used to determine the crystal electric-field (CEF) parameters of the material. The black dashed line shows the best fit to the experimental data ($\chi^2 = 0.17$), considering solely the lowest nondispersing excitation at $E = 12.0(4)$ meV (see also Tabl. I). The background consists of two Gaussians centered at zero energy transfer, representing the elastic line and monotonically decreasing scattering contribution at increasing energy transfers. The red dashed line shows the predicted excitation spectrum without the background model. **b** The inclusion of the peaks at $E = 23(1)$ and 36(1) meV with intensity dominating at $6 < |q| < 10$ Å^{-1}, yields a destabilised fit ($\chi^2 = 58$) and worsens the refinement of the $E = 12.0(4)$ meV transition (see black and red dashed lines). **c** Calculated inverse susceptibility as function of temperature (see Ref.[7] for comparison with earlier refinements and with experimental results).

In a localised moment picture the ground state doublet $|\Psi^{\pm}\rangle$ is split by magnetic long-range order. Thus, the dipole allowed transitions between the ground state wavefunctions give an estimation of the neutron cross-section expected in the ordered state. For YbAgGe we find $\langle\Psi^+|J_x|\Psi^-\rangle = 3.48$, $\langle\Psi^+|J_y|\Psi^-\rangle = 0.0108$ and $\langle\Psi^+|J_z|\Psi^-\rangle = 0$. The strongest component indicates the preferred ordered moment direction which is the local x-axis of the three magnetic Yb sites, *i.e.* (1, 0, 0) for Yb1, (0, 1, 0) for Yb2 and (1, 1, 0) for Yb3 (see also Ref.[3]). The other components give a cross-section estimation for

the magnetic fluctuations perpendicular to the ordered moment. Their small value indicate very weak spin-wave excitations that are expected to be hardly observable in an inelastic neutron scattering experiment.

Supplementary Note 3: Insight into exchange couplings

Despite various contributing ingredients in frustrated metals, it is often possible to determine the main couplings through a localised Heisenberg exchange Hamiltonian. For YbAgGe we used the effective Hamiltonian

$$H = \sum_{ij} J_{ij} \mathbf{S}_i \cdot \mathbf{S}_j + \sum_{ij} J_{cij} \mathbf{S}_i \cdot \mathbf{S}_j + A \sum_i \mathbf{S}_i^2. \tag{1}$$

The first term corresponds to the exchange couplings J_{ij} between spins $\mathbf{S}$ on sites i and j of the distorted Kagome plane. The second term pertains the exchanges with components along the c-axis, J_{cij}, and the last term accounts for the magnetic anisotropy A.

Our experimental findings and CEF considerations suggest that the energy scale of the magnetic anisotropy and exchange couplings are of the same order in YbAgGe. Thus, the anisotropy does not dominate the selection of the ordering vector. We fixed the parameter to A = -1. Another important experimental fact is that the $q_z = \frac{1}{3}$ component of the ordering vector is remarkably persistent in all reported ordered magnetic phases up to $\mu_0 H = 7$ T[3]. This suggests that exchange interactions extending along the c-axis are strong, competing among themselves and with the intraplane couplings.

We minimized the Hamiltonian of Eq.(1) using the SpinW software[8], restricting the J-parameter space to the nearest neighbours presented in Suppl. Fig. 3 and scanned the exchanges within [-1..1] intervals using a grid step of 0.05. We recorded the minimal energy solutions with propagation vectors corresponding to the experimentally observed vectors q_1=($\frac{1}{3}$, 0, $\frac{1}{3}$), its arms and q = (0, 0, $\frac{1}{3}$). Firstly, we considered only exchanges up to the second next-nearest neighbours in- and out- the plane, presented in Suppl. Fig. 3a. The solutions with propagation vectors $q_z = \frac{1}{3}$ spread over all considered intervals of J_1, J_2 and J_{c1}, but J_{c2} was confined to [-0.5..0.5] region (Suppl. Fig. 3c). With this set of couplings the propagation vector q_1=($\frac{1}{3}$, 0, $\frac{1}{3}$) was not found. Then we included the equidistant but distinct J_{3a} and J_{3b} in-plane couplings (Suppl. Fig. 3b) into the simulations. The difference between J_{3a} and J_{3b} becomes apparent when inspecting the hexagon encircled by a dashed black line around the $\bar{6}$-axis (cell origin) in Suppl. Fig. 3b. The cyan colored J_{3a} bonds pass though the middle of the hexagon, while the pink J_{3b} bonds span its periphery. As the J-parameter space expanded significantly, we used a cruder grid step of 0.3. The solutions with q_1=($\frac{1}{3}$, 0, $\frac{1}{3}$) were found for finite J_{3a} and J_{3b} values (both positive and negative). Interestingly, these equidistant, but different couplings were decisive already in selecting the magnetic field-induced states in TmAgGe[9]. For YbAgGe the stabilizing the q_1=($\frac{1}{3}$, 0, $\frac{1}{3}$) propagation vector requires J_{c1} and J_{c2} to be positive, that is AFM (Suppl. Fig. 3d).

Supplementary Note 4: High Field Magnetization and comparison to calculations

The localization degree of the Yb moments was examined through magnetization experiments in pulsed magnetic fields up to $\mu_0 H = 50$ T at the Dresden High Magnetic Field Laboratory, which were normalised to laboratory measurements up to $\mu_0 H = 14$ T reported in Ref.[2]. Supplementary Figure 4 depicts the experimental results measured at $T = 0.53$ K on a single crystal for $H||ab$ (red line) and $H||c$ (orange line), respectively. Overplotted in green and blue are the calculated magnetization curves expected from the CEF scheme. We assign the deviations at low magnetic fields to contributions of the magnetic exchange interactions that were not considered in the calculations. For $H||ab$ we find qualitative agreement in the overall field dependence and quantitative agreement at large magnetic fields. In contrast, qualitative deviations occur for $H||c$ above $\mu_0 H \approx 30$ T. For $H||c$ we find that the crystal was misaligned by $\sim$12° away from the c-axis due to manual alignment of the small crystal in the ^{3}He insert. This misalignment is not relevant for the conclusion that the local moment model cannot account for the experimentally observed magnetization curve.

Supplementary Note 5: Dynamic magnetism in YbAgGe at zero magnetic field

The static and dynamic properties at $\mu_0 H = 0$ T and T = 0.25 - 30 K were studied on CNCS using incident energies E_i = 12, 3.32 and 1.55 meV with elastic energy resolutions ΔE = 1.2, 0.175 and 0.04 meV FWHM. While we show the E_i = 3.32 meV dataset at $T = 0.25$ K in Fig. 3 of the main manuscript, the E_i = 12 and 1.55 meV datasets along (0, 0, L) and (-1/2, 0, L) are shown in Suppl. Fig. 5a and b, respectively. The E_i = 12 meV dataset provides evidence that the column like excitations at (H, 0, 1/3), (H, 0, 2/3) and (0, 0, 1/2) are the only observable magnetic excitations below the first CEF transition at an energy transfer E = 12 meV. The additional cone-like signal around (0, 0, 2) is an acoustic phonon. The high-resolution setting at E_i = 1.55 meV reveals no clear excitation gap at low energy transfer. This is further supported by the one-dimensional energy cuts various reciprocal lattice positions shown in Suppl. Fig. 6a, suggesting that the excitations are quasielastic.

Following reported inelastic neutron scattering experiments at specific (H, 0, 1/3) and (1/3, 0, L) positions[10], we fitted the dynamic susceptibility to a quasielastic Lorentzian $\chi''(q, E) = (E\chi'_q \Gamma_q)/(E^2 + \Gamma_q^2)$. $\chi''(q, E)$ is directly probed by neutron scattering, χ'_q is the real part of the static susceptibility at the wavevector q, and Γ_q is a characteristic energy scale. The

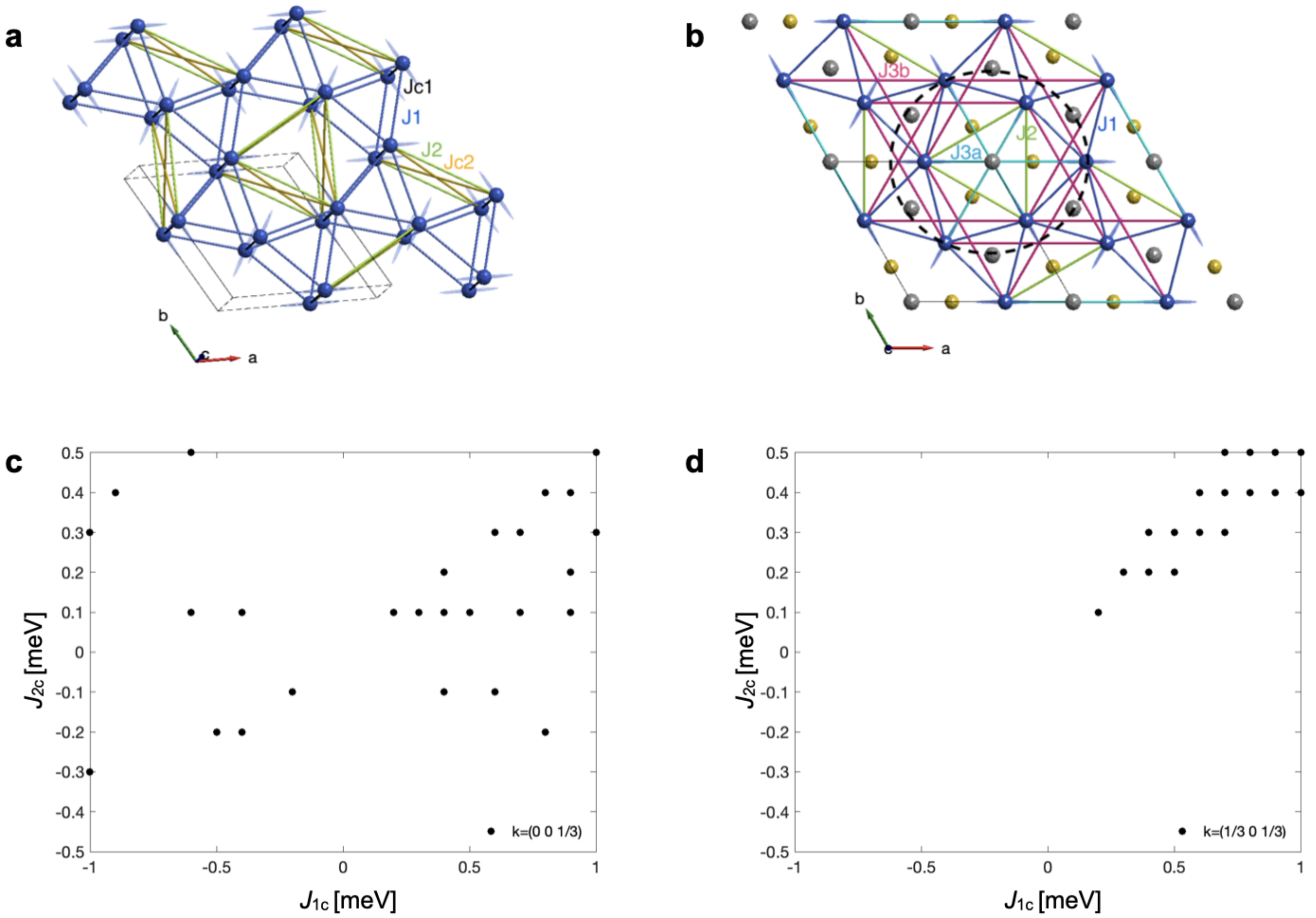

Supplementary Figure 3: **Exchange couplings and results of local Hamiltonian minimization. a** Nearest neighbour exchange couplings J_1 (blue), J_2 (green) in-plane and J_{c1} (black), J_{c2} (gold) out-of-plane alongside with the single-ion anisotropy (blue ellipses). **b** in-plane J_1, J_2, J_{3a} (cyan) and J_{3b} (pink) couplings. Yb, Ge and Ag atoms are colored in blue, gray and gold, respectively. The dashed black circle spans over a hexagon of edge sharing Yb-triangles, the elemental building block of the geometrical Yb^{3+} building block. **c** Distribution of the lowest energy solutions with magnetic ordering vectors q= (0, 0, $\frac{1}{3}$) and **d** q_1= ($\frac{1}{3}$, 0, $\frac{1}{3}$) as a function of J_{c1} and J_{c2}. The other couplings, J_1, J_2, J_{c1} vary within [-1 .. 1] intervals, J_{c2} within [-0.5 .. 0.5].

results are shown in Suppl. Fig. 6b,c. We find a wavevector dependent Γ_q that is smallest at $q = (0, 0, 1/2)$ (0.23(7) meV), 0.41(6) meV at $q = (0, 0, 1/3)$ and 0.54(7) meV in average. Their finite values attest that no quantum critical fluctuations are obscuring the magnetic excitation spectrum at $\mu_0 H = 0$ T. Upon increasing temperature the quasielastic Lorentzian fit shows that the wavevector dependence of the static susceptibility χ'_q is suppressed above T^*. The characteristic energy scale follows the dependence $\Gamma_q(T) = \Gamma_q(0) + \Theta(T - T_s)A\sqrt{T}$ with $A = 0.19(2)$ meVK$^{-1/2}$, and $T_s \approx 5$ K occurring in the step function Θ is close to where χ'_q is maximal.

Supplementary Note 6: Magnetic field dependence of the dynamic properties

The magnetic field dependent excitations were measured on the cold neutron multiplexing spectrometer CAMEA. In Suppl. Fig. 7 we show reciprocal space maps of the (H, H, L) plane at an energy transfer $E = 0.6(1)$ meV at $\mu_0 H =$ 0, 2, 5.5, 11 T. The data were measured with an incident energy $E_i = 5$ meV. The zero field measurements confirm the column-like excitations with spectral weight along $(H, 0, 1/3)$, $(H, 0, 2/3)$ and around $(0, 0, 1/2)$ observed on CNCS. Apart of a decrease of the neutron intensity the excitations remain unchanged until $\mu_0 H = 5.5$ T under a magnetic field along H ||[1-10], despite various changes in the magnetic long-range order[3]. At $\mu_0 H = 11$ T we find a broad dispersion along (0, 0, L) that is quasielastic at (0, 0, 1/3) and (0, 0, 2/3) and maximal at (0, 0, 1) with an energy transfer $E = 4$ meV (see Fig. 5 of the main manuscript).

Supplementary Note 7: Linear Spin-wave simulations

We attempted to model the magnetic excitation spectrum of YbAgGe using a minimal local-moment Hamiltonian. The diffraction experiments in Fig. 2 and 3 of the main manuscript reveal that the correlations length along the c-axis are largely

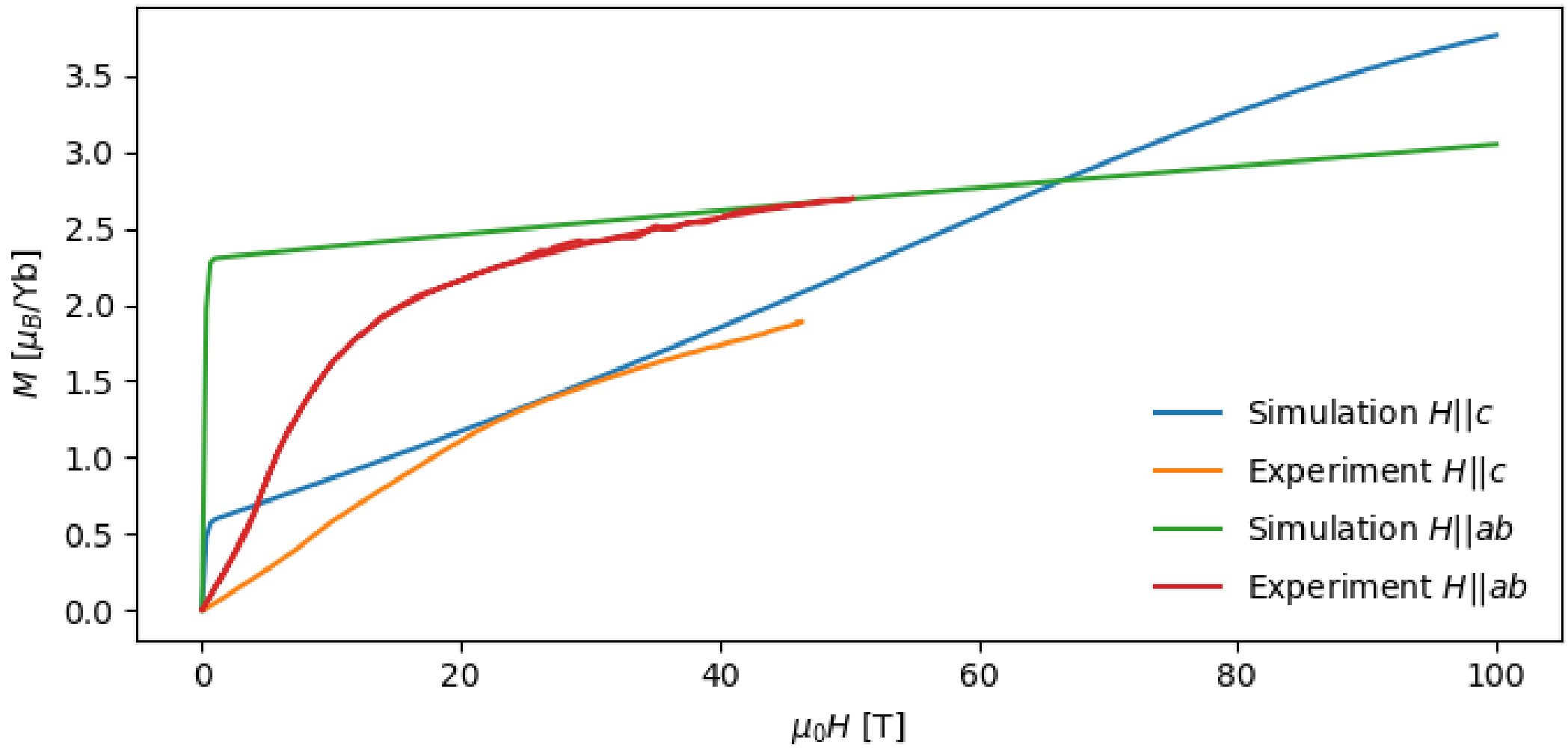

Supplementary Figure 4: High-field magnetization. Field dependent magnetization of YbAgGe for $H||ab$ and $H||c$. The red and orange curves are measurements at $T = 0.53$ K. In green and blue we show the field dependence expected from the crystal electric-field scheme.

exceeding the ones in the Kagome plane. The excitation spectrum shows an energy dependence of the fluctuations that takes place mainly along the L-direction, for which earlier diffraction results reveal a robust $q_z \approx \frac{1}{3}$ component of the magnetic wavevector (see also SI Note1)[3]. Thus, we first looked into one-dimensional Heisnberg chain Hamiltonians with interaction parameters that retain the $q_z \approx \frac{1}{3}$ periodicity and with spin-wave boundaries that broadly reflect the excitation energy range of the $\mu_0 H = 11$ T spectrum shown in Fig. 5 of the main manuscript

$$H = \sum_{ij} J_{cij} \mathbf{S}_i \cdot \mathbf{S}_j + A \sum_i \mathbf{S}_i^2 - g\mu_B \mu_0 \mathbf{H} \sum_{\mathbf{i}} \mathbf{S_i}. \tag{2}$$

The first term describes the exchange couplings along the c-axis between spins $\mathbf{S}$ on sites i and j with exchange couplings J_{cij}. The second term accounts for the anisotropy A, and the last term is the Zeeman term with $H||[1, -1, 0]$. We assumed independent chains along the c-axis with two nearest-neighbour interactions J_{c1}, and J_{c2}, whose ratio leads to a magnetic structure with a propagation vector retaining the $q_z \approx \frac{1}{3}$ periodicity. The values were adjusted such that a spin-wave boundary of $E \sim 3.5$ meV was obtained at $\mu_0 H = 11$ T, in accordance to the experimental data (see Fig. 5 of the main manuscript).

Supplementary Figure 8a shows the spin-wave dispersion along $(0, 0, L)$ for the Heisenberg chain with antiferromagnetic interactions $J_{c1} = 1.5$ meV, $J_{c2} = 0.6$ meV and $A = 0$ at zero magnetic field. The configurations yield a spiral structure with magnetic Goldstone modes within $E = 0$ - 2 meV. Both, the finite axial anisotropy $A < 0$ mimicking the crystal-field anisotropy and non-zero magnetic field lead to a gaped excitation spectrum, as shown in Suppl. Figs. 8b, c for $A =$ -0.1 meV and $\mu_0 H = 11$ T, respectively. This contrasts the experimental observations, which report soft excitations up to $\mu_0 H = 11$ T. Thus, we concluded that a one-dimensional Heisenberg chain does not provide a satisfactory description of the observed magnetic excitation spectrum. In an alternative approach we incorporated the crystal-field anisotropy via anisotropic exchange interactions using the effective Hamiltonian

$$H = \sum_{ij} J_{cij}^{xx} S_i^x \cdot S_j^x + J_{cij}^{yy} S_i^y \cdot S_j^y + J_{cij}^{zz} S_i^z \cdot S_j^z \tag{3}$$

with $J_{c1}^{xx} = J_{c1}^{yy} = 0.2$, $J_{c1}^{zz} = 1.5$, $J_{c2}^{xx} = J_{c2}^{yy} = 0$ and $J_{c2}^{zz} = 0.6$ meV. The model yields a magnetic structure with $q_z \approx \frac{1}{3}$. The resulting spin waves are displayed in Suppl. Fig. 8d, which is also inconsistent with the experimental observations.

Supplementary References

* Electronic address: daniel.mazzone@psi.ch
† Electronic address: oksana.zaharko@psi.ch

1 P. C. Canfield, Reports on Progress in Physics **83**, 016501 (2019).
2 E. Morosan, S. Bud'ko, P. Canfield, M. Torikachvili, and A. Lacerda, Journal of Magnetism and Magnetic Materials **277**, 298 (2004).

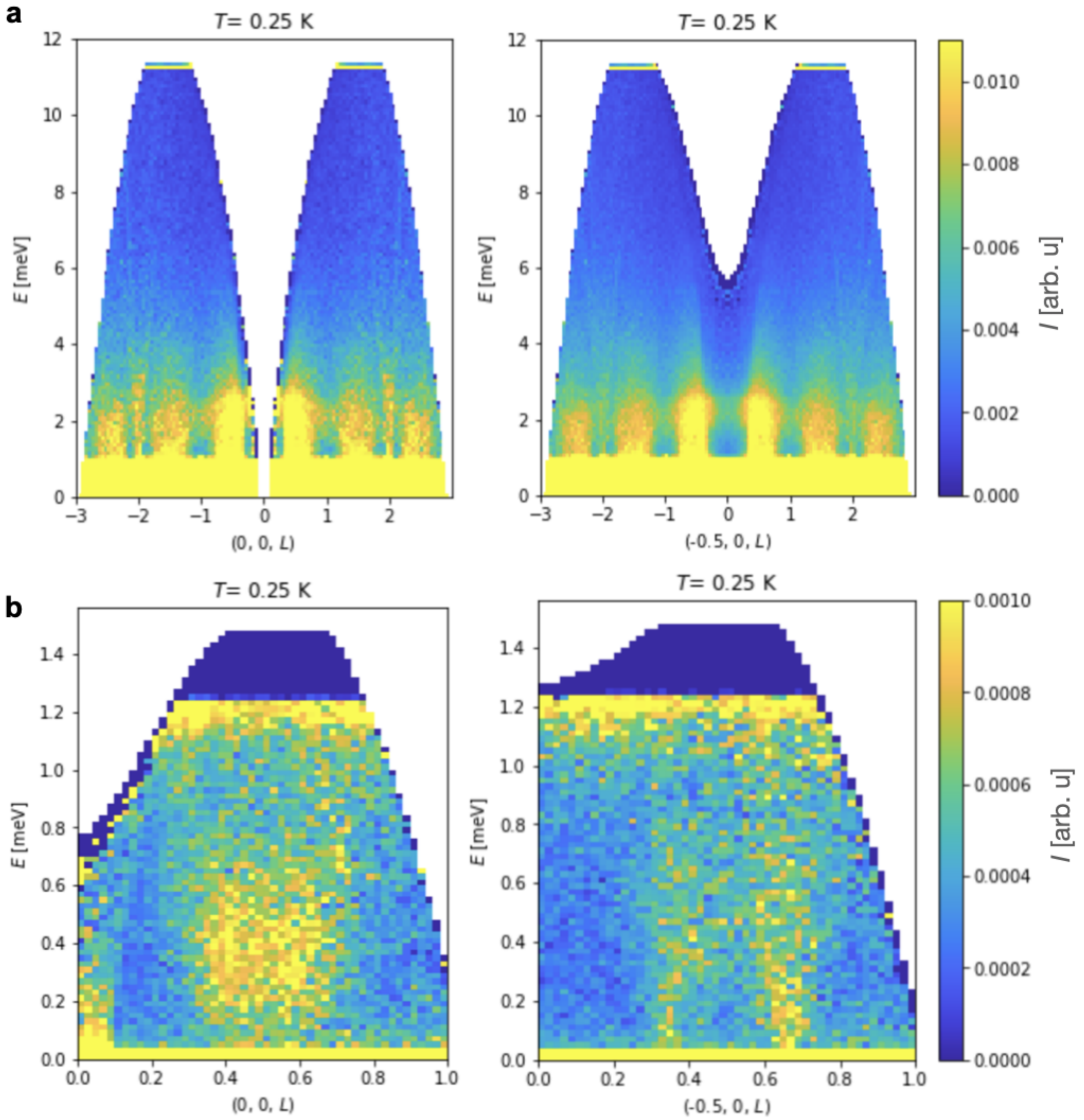

Supplementary Figure 5: **Magnetic excitations at** $\mu_0 H = 0$ **T.** Magnetic excitation spectrum of YbAgGe along (0, 0, L) and (-1/2, 0, L) measured at $T = 0.25$ K and $E_i = 12$ and 1.55 meV in **a** and **b**, respectively. We used a reciprocal space integration range $\Delta Q = [-0.2, 0.2]$ perpendicular to the cutting direction.

[3] C. B. Larsen, E. Canévet, D. G. Mazzone, C. Rüegg, B. Fåk, D. F. McMorrow, E. Ressouche, G. J. McIntyre, S. L. Bud'ko, P. C. Canfield, and O. Zaharko, Phys. Rev. B **104**, 054424 (2021).

[4] S. L. Bud'ko, E. Morosan, and P. C. Canfield, Phys. Rev. B **69**, 014415 (2004).

[5] G. M. Schmiedeshoff, E. D. Mun, A. W. Lounsbury, S. J. Tracy, E. C. Palm, S. T. Hannahs, J.-H. Park, T. P. Murphy, S. L. Bud'ko, and P. C. Canfield, Phys. Rev. B **83**, 180408 (2011).

[6] A. Scheie, Journal of Applied Crystallography **54**, 356 (2021).

[7] T. Matsumura, H. Ishida, T. J. Sato, K. Katoh, Y. Niide, and A. Ochiai, Journal of the Physical Society of Japan **73**, 2967 (2004), https://doi.org/10.1143/JPSJ.73.2967 .

[8] S. Toth and B. Lake, J. Phys.: Condens. Matter **27**, 166002 (2015).

[9] C. B. Larsen, D. G. Mazzone, N. Gauthier, H. D. Rosales, F. A. Gómez Albarracín, J. Lass, X. Boraley, S. L. Bud'ko, P. C. Canfield, and O. Zaharko, Phys. Rev. B **107**, 224419 (2023).

[10] B. Fåk, D. F. McMorrow, P. G. Niklowitz, S. Raymond, E. Ressouche, J. Flouquet, P. C. Canfield, S. L. Bud'ko, Y. Janssen, and M. J. Gutmann, Journal of Physics: Condensed Matter **17**, 301 (2004).

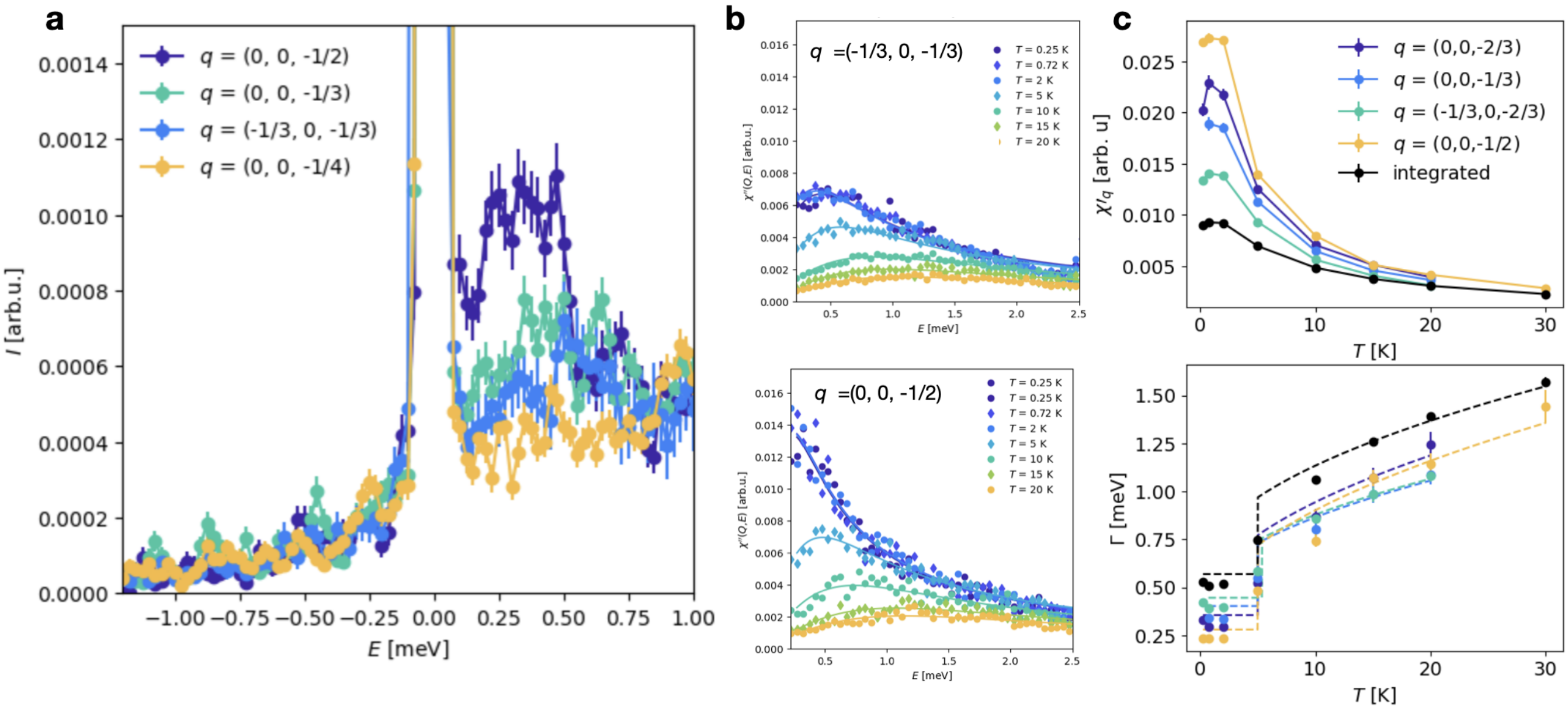

Supplementary Figure 6: **Temperature dependence of the dynamic fluctuations.** **a** One dimensional line cuts through various reciprocal lattice positions at $T = 0.25$ K using $E_i = 1.55$ meV with an integration of ΔQ = [-0.05, 0.05]. **b** One dimensional line cuts through $q = (0, 0, \text{-}1/2)$ and (-1/3, 0, -1/3) as function of temperature using E_i= 3.32 meV with an integration range of ΔQ = [-0.05, 0.05] around the reciprocal lattice positions. Overplotted (dashed lines) are fitted quasielastic Lorentzian lineshapes $\chi''(q, E) = (E\chi'_q\Gamma_q)/(E^2+\Gamma_q^2)$. $\chi''(q, E)$ is the imaginary part of the dynamic susceptibility directly revealed by neutron scattering, χ'_q is real part of the static susceptibility at the wavevector q, and Γ_q is a characteristic energy scale. The temperature dependence of χ'_q and Γ_q for different qs are shown in **c**. The characteristic energy scale follows the dependence $\Gamma_q(T) = \Gamma_q(0) + \Theta(T - T_s)A\sqrt{T}$ with $A = 0.19(2)$ meVK$^{-1/2}$, and $T_s \approx 5$ K occurring in the step function Θ is close to where χ'_q is maximal.

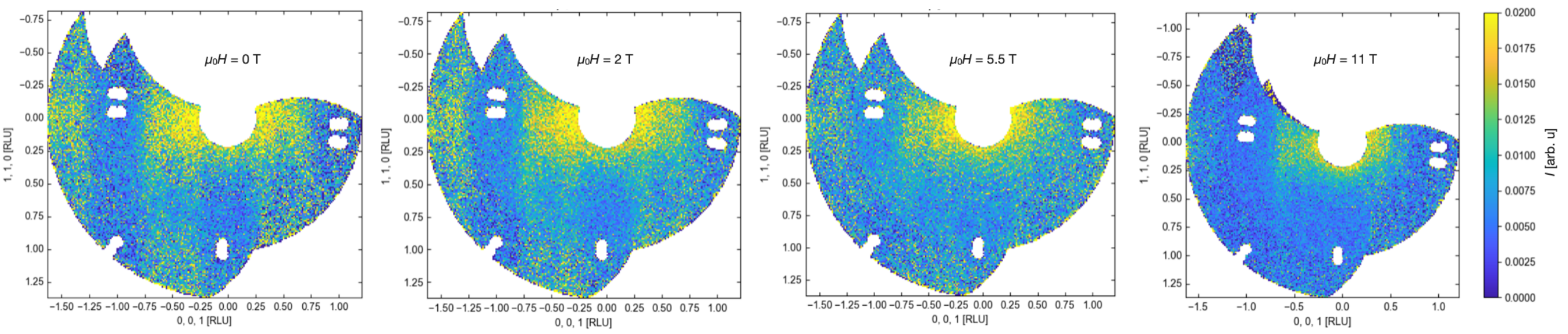

Supplementary Figure 7: **Field dependence of excitations.** Reciprocal space maps of the (H, H, L) plane at an energy transfer $E = 0.6$ meV at $\mu_0 H$ = 0, 2, 5.5, 11 T along H ||[1-10]. The data were measured with E_i = 5 meV using an energy integration range ΔE = [0.5, 0.7] meV.

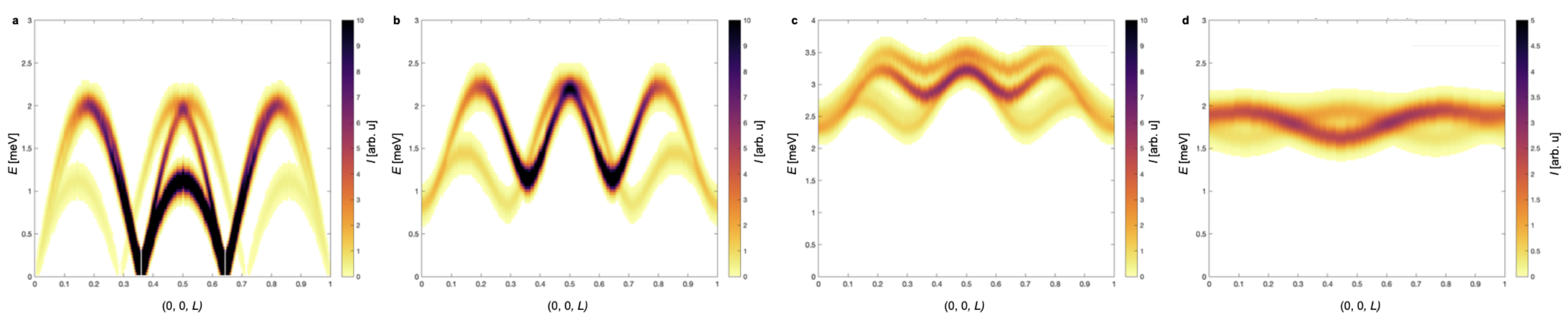

Supplementary Figure 8: **Linear spin-wave simulation.** Simulation of the magnetic excitation spectrum along $(0, 0, L)$ using a one dimensional Heisenberg chain without local anisotropy in **a**, with a small axial anisotropy in **b**, and under magnetic field along $H||[1, -1, 0]$ in **c**. Subpanel **d** shows a simulation of a xxz-chain, in which the crystal-field anisotropy was incorporated via anisotropic exchange interactions.
